# TLR7 alters the maternal immune landscape during influenza A infection to increase maternal and fetal morbidity

**DOI:** 10.1126/sciadv.ady2382

**Published:** 2026-04-29

**Authors:** Gemma S. Trollope, Mark A. Miles, Madison Coward-Smith, Felicia Liong, Doug A. Brooks, John J. O’Leary, Stavros Selemidis, Stella Liong

**Affiliations:** ^1^ Centre for Respiratory Science and Health, School of Health and Biomedical Sciences, RMIT University, Bundoora, Victoria 3083, Australia.; ^2^ School of Pharmacy and Biomedical Sciences, College of Health, Adelaide University, North Terrace, Adelaide, South Australia 5001, Australia.; ^3^ Discipline of Histopathology, School of Medicine, Trinity Translational Medicine Institute (TTMI), Trinity College Dublin, Dublin, Ireland.

## Abstract

Pregnant women infected with influenza A virus (IAV) are at increased risk of severe disease, leading to maternal and fetal complications. Toll-like receptor 7 (TLR7) recognizes single-stranded RNA viruses, including IAV, yet its role in maternal immune responses and pregnancy outcomes during IAV infection is poorly understood. Here, we demonstrate that TLR7-knockout (TLR7^−/−^) pregnant mice showed reduced disease severity, despite similar pulmonary viral titers to wild-type (WT) mice. TLR7^−/−^ dams exhibited distinct pulmonary responses, including reduced lymphocyte infiltration, enhanced neutrophil response, and a shift from type I to type II interferon activity. TLR7 signaling was found to be essential for the development of IAV-induced vascular dysfunction during pregnancy. Offspring from TLR7^−/−^ mice showed improved body weight and reduced placental and fetal brain inflammation compared to WT counterparts. We provide evidence that TLR7 is a critical mediator of adverse pregnancy outcomes during IAV infection and a potential therapeutic target to reduce maternal and fetal morbidity.

## Teaser

TLR7 signaling drives systemic maternal and fetal pathology during gestational influenza infection.

## INTRODUCTION

Influenza viruses remain a major global health care burden. Despite largely being considered a self-limiting infection, influenza A viruses (IAVs) cause ~1 billion annual global infections, with 3 to 5 million cases considered severe. These severe cases contribute to 290,000 to 650,000 deaths worldwide, mostly due to respiratory distress (*1*). The risk for influenza-related morbidity and mortality is substantially greater for at-risk populations, particularly for pregnant women and their offspring. During the 1918 H1N1 “Spanish flu” pandemic, the mortality rate for pregnant women was 27% compared to just 1% in age-matched nonpregnant women (*2*, *3*). The risk of mortality also increases with gestational age, with rates of 7.1, 26.8, and 64.3% across the first, second, and third trimesters, respectively (*4*). Pregnant women with influenza experience more severe outcomes including increased rates of hospitalization, intensive care unit (ICU) admission, and mechanical ventilation (*5*). From the perspective of the fetus, maternal influenza infection can lead to a range of acute and long-term health complications. These are likely driven by aberrant maternal immune activation (*6*) rather than direct viral transmission, as vertical transmission is rare (*7*, *8*). These complications for the fetus include increased risk of spontaneous abortion (*9*), preterm birth (*10*), neurodevelopmental changes (*11*), and congenital defects including neural tube defects and congenital heart defects (*12*).

Using preclinical mouse models, we have previously shown that influenza infection during pregnancy increases risk of systemic disease, with the maternal aorta identified as a previously unrecognized extra-pulmonary site of IAV RNA detection (*13*). IAV mRNA is detectable in the aorta as early as 24 hours postinfection and at 3 days postinfection (dpi) the IAV viral RNA levels in the aorta were 10-fold higher in pregnant dams compared to nonpregnant IAV-infected female mice (*13*). This aortic viral RNA signal coincides with the development of a peripheral “vascular storm” a state of maternal vascular dysfunction characterized by impaired endothelial relaxation and excessive immune cell infiltration (including neutrophils, CD4^+^ and CD8^+^ T cells, and Ly6C^low^ and Ly6C^high^ monocytes), which mediate a strong pro-inflammatory and antiviral response (*13*). This maternal “vascular storm” leads to suboptimal blood flow to the fetal-placental unit, impairing fetal development and growth. However, the mechanisms that underlie this IAV-driven “vascular storm” event(s) remain unclear.

Toll-like receptor 7 (TLR7) is a primary sensor for RNA viruses like IAV and has recently emerged as a key factor in immunopathologies, including those that occur during pregnancy. TLR7 activation in response to viral infection triggers a potent innate immune response, but how it mediates severe maternal and fetal outcomes during influenza infection remains unclear. Previous studies have suggested that TLR7 activation alone can induce maternal and offspring pathologies (*14*–*16*). Furthermore, we have demonstrated that TLR7 expression is significantly up-regulated in the maternal aorta during gestational IAV infection (*13*). In this study, we further investigate the role of TLR7 in modulating maternal and fetal outcomes during gestational IAV infection. By using TLR7^−/−^ mice, we show that the absence of TLR7 results in reduced maternal morbidity, despite similar viral loads in the lungs, suggesting that TLR7-driven immune responses are crucial for the observed severe disease outcomes. We find that TLR7 deletion alters immune cell infiltration in both the lungs and aorta, reduces vascular dysfunction, and prevents fetal growth restriction. These findings implicate TLR7 as a central mediator of the pathological “vascular storm” in pregnant mice and provide insight into how TLR7 may exacerbate both maternal and fetal disease during influenza infection.

## RESULTS

### IAV disease morbidity is reduced in TLR7^−/−^ pregnant mice

**Fig. 1.**
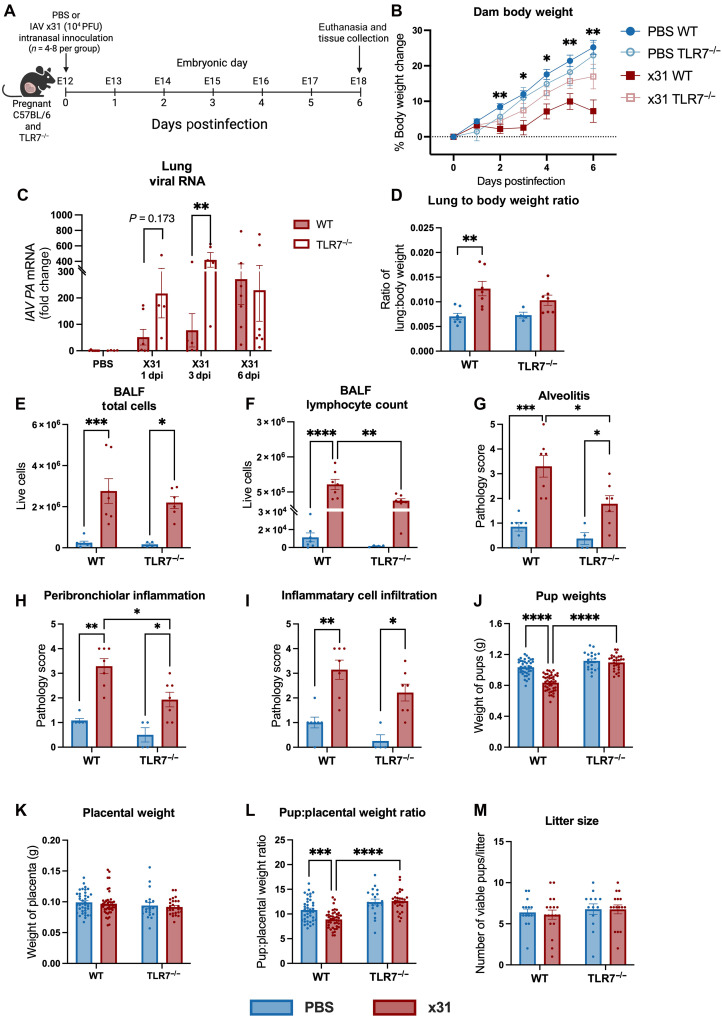
TLR7 deletion reduces IAV-induced pregnancy perturbations. Pregnant WT C57Bl/6 and TLR7-knockout (TLR7^−/−^) were inoculated with PBS or HKx31 (x31; 10^4^ PFU) to assess disease severity at 6 dpi. (**A**) Schematic of the experimental timeline [Created in BioRender. Trollope, G. (2026) https://BioRender.com/k11s712]. (**B**) Change in body weight from 0 to 6 dpi (PBS WT versus x31WT). (**C**) IAV polymerase transcripts were quantified by qPCR at days 1, 3, and 6 dpi and are presented as the fold change in expression from uninfected controls relative to *Rps18*. (**D**) Pulmonary edema was assessed on lung–to–body weight ratio to standardize the level of edema based on body weight. Pulmonary inflammation was assessed by counting the total number of live cells (**E**) in the BALF and reporting proportions of cells including (**F**) lymphocyte numbers. The histopathology of infected lungs was assessed on the basis of the criteria of (**G**) alveolitis, (**H**) peribronchiolar inflammation, and (**I**) immune cell infiltration. E18 fetal development was evaluated by assessing the (**J**) gross body weight, (**K**) gross placental weights, (**L**) pup–to–placental weight ratio, and (**M**) number of viable fetuses per litter, which were also compared to rule out any confounding implications on changes in maternal or fetal weight following infection. Statistical analysis was determined via two-way repeated measure ANOVA with Geisser-Greenhouse’s epsilon correction [*F*(1.494,32.88) = 113.1] (B), two-way ANOVA with Tukey’s post hoc test [(C) to (F) and (J) to (L)], or Mann-Whitney *U* test [(G) to (I) and (M)] (*P ≤ 0.05; **P ≤ 0.01; ***P ≤ 0.001; ****P ≤ 0.0001). All data are presented as means ± SEM; [(B) to (I)] *n* = 4 to 7, [(J) to (L)] *n* = 20 to 44, and (M) *n* = 13 to 18 per group.

Age-matched (8 to 12 weeks old) pregnant wild-type (WT) C57BL/6 and TLR7-knockout (TLR7^−/−^) mice were inoculated with 10^4^ plaque-forming units (PFU) of the IAV strain, A/HKx31 (x31) at midpregnancy, on embryonic day 12.5 (E12.5) (Fig. 1A). Using body weight as an indicator of disease morbidity, uninfected WT mice gained ~25% body weight from E12.5 to E18.5 gestation (equivalent to early term gestation in human pregnancies). In contrast, x31 infected WT mice showed blunted body weight gain, averaging 12% from E12.5 to E18.5 gestation. In x31 infected TLR7^−/−^ mice, body weight gain was similar to uninfected TLR7^−/−^ and WT controls, with an average gain of 20% (Fig. 1B). To examine whether differences in pulmonary viral burden contributed to disease severity, we quantified viral titers at 1, 3, and 6 dpi. Viral kinetics were comparable between genotypes at 1 dpi. By 3 dpi, TLR7^−/−^ dams displayed a modest but statistically significant increase in pulmonary viral titers relative to WT dams; however, by 6 dpi, viral titers had equalized, and no differences were detectable between strains (Fig. 1C). These data indicate that, although early viral replication may be slightly higher in TLR7^−/−^ mice, overall pulmonary viral burden converges over the course of infection, supporting the conclusion that differential disease severity reflects immune pathology rather than sustained differences in viral clearance. Future studies incorporating immunohistochemistry or in situ hybridization across distinct epithelial compartments of the respiratory tract will be important to comprehensively define the spatial dynamics of viral replication, although this lies beyond the scope of the current study.

As a primary respiratory pathogen, influenza can cause pneumonia, which poses a particular risk during pregnancy, given that pneumonia is the leading nonobstetric cause of death among pregnant women (*17*). Pulmonary edema at 6 dpi was measured as another surrogate marker of disease severity, with gross lung weights and lung–to–body weight ratios often used as a proxy for pulmonary edema (*18*, *19*). Both x31 infected WT and TLR7^−/−^ mice displayed increased gross lung weights compared to their uninfected counterparts (fig. S1A). However, when normalized to body weight (lung–to–body weight ratio), only x31 infected WT mice showed significant increases in lung weights relative to body weight when compared to their uninfected counterparts (Fig. 1D). Analysis of airway inflammation during x31 infection revealed no significant differences in the total number of immune cells infiltrating the airways, as measured from the bronchoalveolar lavage fluid (BALF) (Fig. 1E). Although total immune cell infiltration was similar between infected WT and TLR7^−/−^ mice, the composition of infiltrating cells differed (fig. S1B). Specifically, TLR7^−/−^ mice showed a significant reduction in the number of lymphocytes in the BALF compared to infected WT mice (Fig. 1F) with no alterations in macrophage or neutrophil absolute numbers (fig. S1, C and D).

We next sought to characterize the role of TLR7 on pulmonary disease progression during gestational IAV infection. In pregnant WT mice, x31 infection was associated with alveolitis, peribronchiolar inflammation and inflammatory cell infiltration in the lungs (Fig. 1, G to I). Comparatively, infected TLR7^−/−^ mice displayed significantly reduced alveolitis severity and peribronchiolar inflammation when compared to infected WT mice (Fig. 1, G and H). Consistent with the BALF data (Fig. 1E), loss of TLR7 did not change the total inflammatory cell infiltration within the lungs during gestational IAV infection (Fig. 1I).

To assess whether this reduced disease morbidity observed in infected TLR7^−/−^ dams extended to improved offspring health in utero, fetal and placental weights were measured at 6 dpi. As expected, x31 WT offspring were significantly smaller than offspring from uninfected WT dams. In the absence of TLR7, fetal growth was no longer adversely affected by maternal x31 infection (Fig. 1J), with offspring from both infected and uninfected TLR7^−/−^ mice having similar body weights in utero. Moreover, x31 TLR7^−/−^ offspring were found to be significantly heavier than x31 WT offspring (Fig. 1K). Although gross placental weight remained unchanged across all groups (Fig. 1K), given the differences in pup weights, the pup–to–placental weight ratio was significantly smaller in the infected WT compared to TLR7^−/−^ offspring (Fig. 1L). Of note, litter sizes (viable pups, excluding resorptions) in utero were comparable across all groups (Fig. 1L), and controlling for litter size as a random effect variable was shown not to influence fetal weight or pup–to–placental weight ratio (*P* = 0.0852 and *P* = 0.6339, respectively). Therefore, the TLR7-dependent changes in dam and offspring body weights during infection were not due to litter size effects. Additional E18 fetal biometrics analysis was recorded in a separate cohort of offspring litters, and we found no differences in number of fetal resorptions per litter nor any differences in placental diameters across all groups; however, fetal crown-to-rump length and abdominal girths of E18 TLR7^−/−^ offspring were significantly smaller than WT offspring (fig. S1, E to H). Although x31 infection significantly reduced abdominal girth circumferences in both TLR7^−/−^ and WT offspring, x31 infection did not affect pup lengths. Collectively, these findings indicate that, despite no differences in lung viral titers of WT and TLR7^−/−^ pregnant mice, overall maternal and fetal morbidity during gestational IAV infection is reduced in pregnant TLR7^−/−^ compared to WT mice.

### Loss of TLR7 alters lung immune cell composition and promotes a stronger pulmonary type II interferon response

**Fig. 2.**
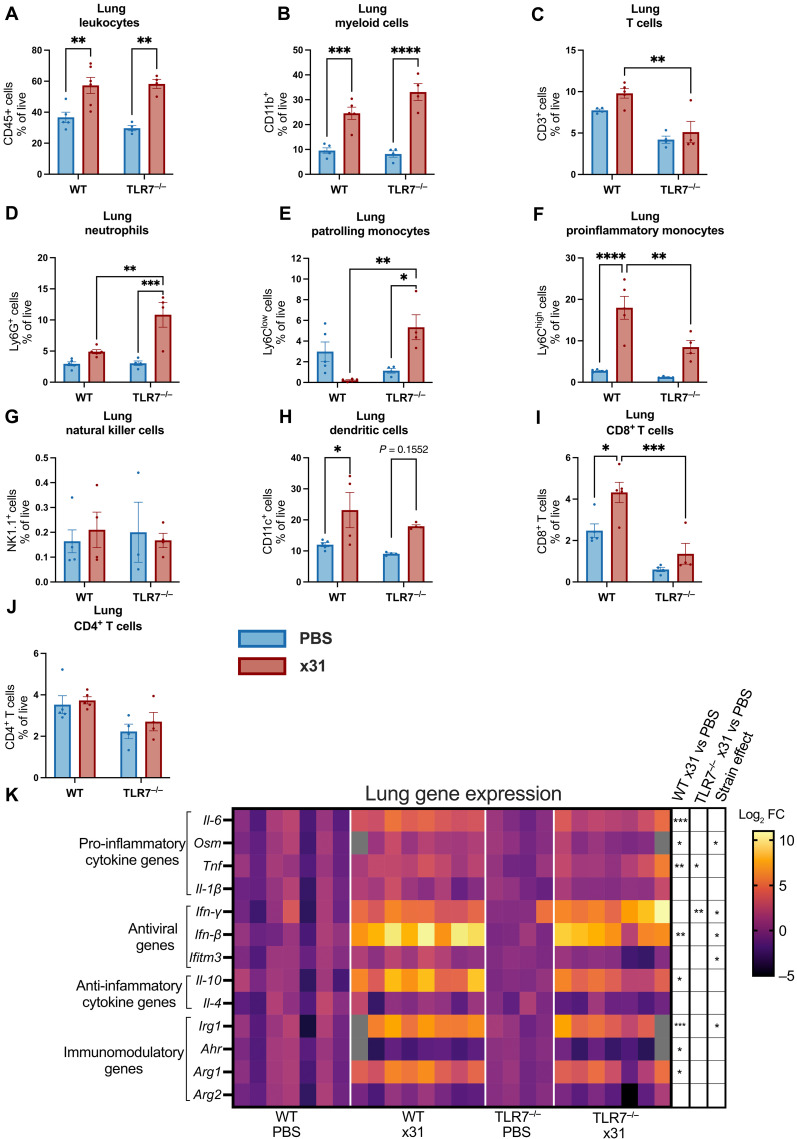
TLR7 deletion alters pulmonary immune cell infiltration and inflammatory gene expression during gestational IAV infection. (**A** to **J**) Lung cell suspensions from infected and uninfected WT and TLR7^−/−^ mice were phenotyped by flow cytometry. (**K**) qPCR analysis of pro- and anti-inflammatory cytokine, antiviral, and immunomodulatory genes was normalized to the expression of *Rps18* and expressed as a fold change (FC) relative to PBS controls. Gray box indicates that the sample was missing from this gene analysis. Strain effect denotes statistical significance between infected genotypes (x31 WT versus x31 TLR7^−/−^). Statistical analysis was determined via two-way ANOVA with Tukey’s post hoc test for multiple comparison (*P ≤ 0.05; **P ≤ 0.01; ***P ≤ 0.001; ****P ≤ 0.0001). All data are presented as means ± SEM; [(A) to (J)] *n* = 4 to 5 and (K) *n* = 4 to 8 per group.

Given the differences in BALF immune cellularity between IAV-infected pregnant TLR7^−/−^ and WT mice, we next sought to elucidate how TLR7 modulates immune cell recruitment into the lungs. Supporting the immune cell infiltration data (Fig. 1I), we found that, in response to x31 infection, both TLR7^−/−^ and WT mice had similar increases in the frequency of live, CD45^+^ leukocytes, and CD11b^+^ myeloid cells (Fig. 2, A and B; gating strategy: fig. S2). Loss of TLR7, however, revealed distinct changes in immune cell infiltration; again, consistent with the BALF data, we found a reduction in the frequency of infiltrating T cells (Fig. 2C) and changes in myeloid subpopulations in the lungs during x31 infection. Specifically, pregnant TLR7^−/−^ mice showed increased infiltration of Ly6G^+^ neutrophils, and Ly6C^low^ patrolling monocytes (Fig. 2, D and E), and reduced number of pro-inflammatory Ly6C^high^ monocytes compared to infected WT mice, although no alteration in natural killer (NK) cell infiltration in the lungs was observed (Fig. 2, F and G). In addition, whereas x31 infection triggered significantly increased infiltration of CD11c^+^ dendritic cells (DCs) into the lungs of pregnant WT mice, this DC response was not significant in infected pregnant TLR7^−/−^ mice (*P* = 0.1552; Fig. 2H). T cell analysis in the lungs revealed that CD8^+^ T cells accounted for the increased T cell infiltration in WT mice, with higher frequencies of live CD8^+^ observed in infected WT mice compared uninfected WT and infected TLR7^−/−^ mice (Fig. 2I) although no significant change in CD4^+^ infiltration between was observed across any group (Fig. 2J).

We next examined the pro-inflammatory immune responses in the lungs following gestational IAV infection. Infection of pregnant WT mice resulted in significantly increased cytokine expression. As expected, gestational IAV infection drove the increased expression of several pro-inflammatory cytokines in the lungs of WT-infected mice [Fig. 2K (heatmap) and fig. S3 (individual graphs)]. However, when comparing the expression profiles after IAV infection, we found that TLR7^−/−^ mice displayed significantly reduced levels of the Oncostatin M (*Osm*) gene, interferon (IFN)–induced transmembrane protein 3 gene (*Ifitm3*), and immune-responsive gene 1 (*Irg1*) compared to infected WT dams. We also assessed the gene expression of antiviral IFN type I (*Ifn-*β) and type II (*Ifn-*γ) in the lungs. Pregnant WT mice infected with x31 showed significantly heightened *Ifn-*β responses compared to both uninfected WT mice and infected TLR7^−/−^ mice (Fig. 2K and fig. S3). In contrast to infected WT mice, x31 infection was not found to induce a significant *Ifn-*β response in pregnant TLR7^−/−^ mice (*P* = 0.326). Further analysis of type II IFN responses revealed no significant increase in *Ifn-*γ expression following x31 infection in WT mice, whereas TLR7^−/−^ mice displayed a more robust *Ifn-*γ expression to infection compared to WT mice. To determine whether gestational IAV infection elicits a systemic inflammatory response, plasma cytokines and chemokines were quantified at 6 dpi (fig. S4). Infection of pregnant WT dams led to significant increases in circulating interleukin-6 (IL-6), tumor necrosis factor–α (TNFα), and CXCL10 compared with uninfected WT controls. Similar elevations of these cytokines were detected in infected TLR7^−/−^ dams, indicating that systemic pro-inflammatory induction occurs independently of TLR7. In contrast, plasma levels of CCL2, IFN-γ, CCL5, IL-1β, and CXCL2 were not significantly altered by infection in either genotype. Together, these findings demonstrate that, although systemic inflammation accompanies gestational IAV infection, TLR7 primarily regulates downstream tissue-specific immune pathology rather than the magnitude of circulating cytokine induction.

### TLR7 promotes cardiovascular dysfunction and aortic viral RNA during gestational IAV infection

**Fig. 3.**
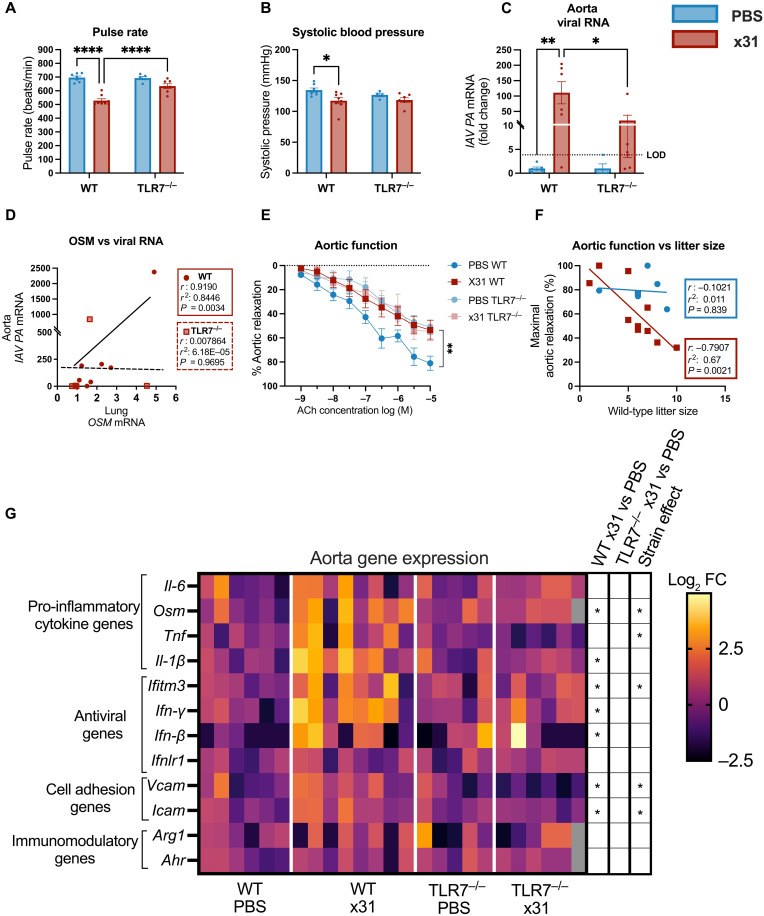
TLR7 deletion limits aortic viral RNA detection and protects from cardiovascular dysfunction during gestational IAV infection. Pregnant WT C57Bl/6 and TLR7-knockout (TLR7^−/−^) were inoculated with PBS or HKx31 (x31; 10^4^ PFU) to assess how gestational influenza infection affects the cardiovascular system. The average (**A**) pulse rate and (**B**) systolic blood pressure were measured over 3 days (4 to 6 dpi). (**C**) IAV polymerase transcripts were quantified by qPCR and presented as fold change relative to uninfected controls, normalized to Rps18. The limit of detection (LOD) is determined as the highest relative ratio observed in uninfected (PBS) control samples. (**D**) The relationship between aortic viral RNA detection and pulmonary Osm expression was assessed by correlation analysis. (**E**) Aortic function was assessed by wire myography and expressed as the capacity for the vessel to relax in response to ACh stimulation (**F**) and was correlated with the size of the litter of WT mice. (**G**) qPCR analysis of pro- and anti-inflammatory cytokine, antiviral, and immunomodulatory genes was normalized to the expression of *Rps18* and expressed as the binary logarithm (log_2_) of the fold change of the geometric mean of the PBS controls. Gray box indicates the sample was missing from this gene analysis. Statistical analysis was determined via two-way ANOVA with Tukey’s post hoc test for multiple comparisons [(A) to (C), (E), and (G)]. Strain effect denotes statistical significance between infected genotypes (x31 WT versus x31 TLR7^−/−^) (G) or Pearson’s correlative analysis [(D) and (F)] (*P ≤ 0.05; **P ≤ 0.01; ****P ≤ 0.0001). All data are presented as means ± SEM; [(A), (B), and (G)] *n* = 5 to 8, [(C) and (D)] *n* = 4 to 8, (E) *n* = 5 to 7, and (F) *n* = 7 to 12 per group.

Given that IAV can disseminate to the aorta and induce vascular dysfunction during pregnancy (*20*), we next sought to investigate the role of TLR7 in viral driven cardiovascular pathology. IAV has been linked to altered blood pressure, cardiac arrhythmias, and conductive abnormalities, with bradycardia or tachycardia being the most common symptoms (*21*, *22*). In our study, pregnant WT mice infected with x31 showed significant bradycardia and reduced systolic blood pressure at 6 dpi (Fig. 3, A and B). In contrast, infected TLR7^−/−^ mice appeared resistant to these IAV-induced cardiovascular effects. Although a modest decrease in diastolic pressure was observed in response to infection in both genotypes, this did not affect mean arterial pressure in TLR7^−/−^ pregnant mice (fig. S5, A and B).

To investigate whether the IAV-induced arrythmia observed in WT pregnant mice was driven by cardiac tissue damage, we assessed the levels of cardiac troponin III in plasma, a cardiac muscle protein, which is only released into circulation upon cardiac cell death. Assessment of IAV-induced cardiac damage at 6 dpi revealed significant up-regulation of circulating cardiac troponin III that was independent of TLR7 (fig. S4C). In contrast to the lungs, IAV-induced cardiac inflammation was not observed at 6 dpi (fig. S5E).

Analysis of viral RNA revealed significantly lower aortic viral transcript in infected TLR7^−/−^ mice compared to WT mice when expressed as fold change relative to uninfected controls (Fig. 3C). Although absolute viral RNA levels were low, ΔCt-based analyses showed consistent genotype-dependent patterns (fig. S10). Osm, a member of the IL-6 family of cytokines is known to drive epithelial barrier disruption in the context of various pulmonary pathologies, including influenza (*23*–*25*). We therefore sought to determine whether *Osm* expression was related to the increased systemic viral burden associated with pregnancy. Correlation analysis of lung cytokine gene expression showed a strong positive relationship between pulmonary *Osm* expression and aortic viral titers in WT mice (*r* = 0.919; Fig. 3D), indicating an association between pulmonary inflammatory signaling and extra-pulmonary viral RNA.

Wire myography was performed on aortic rings to assess vascular function following IAV infection. IAV infection of pregnant WT mice led to impaired endothelial-dependent vasodilation in response to acetylcholine (ACh) in the aorta, compared to uninfected WT mice (Fig. 3E). Although uninfected TLR7^−/−^ mice displayed baseline impaired vasodilation (~50% maximum relaxation) compared to WT mice (~80% maximum relaxation), x31 infection did not further impair this ACh-induced vasodilatory response. Further analysis revealed a negative correlation between litter size and aortic function in WT mice, suggesting that increased cardiovascular demand exacerbates endothelial dysfunction during infection (Fig. 3F), a relationship that was absent in TLR7^−/−^ mice (fig. S5D). Together, these findings indicate that TLR7 plays a central role in regulating endothelial dysfunction during gestational IAV infection, with genotype-dependent differences in aortic viral RNA occurring in the context of low-level extra-pulmonary viral signal rather than overt vascular infection.

### TLR7 is required for IAV-induced vascular inflammation and promotes T cell recruitment to the aorta in pregnant mice

We next sought to assess the role of TLR7 in the aortic pro-inflammatory response during gestational IAV infection. Pro-inflammatory cytokines *Il-1*β and *Osm* were up-regulated in x31 infected WT mice but not in TLR7^−/−^ mice [Fig. 3G (heatmap) and fig. S6 (individual graphs)]. Assessment of the IFN responses in the aorta revealed up-regulated expression of *Ifn-*β, *Ifn-*γ, and *Ifitm3* in x31 infected WT mice, whereas x31 infection in TLR7^−/−^ mice showed no change in IFN responses when compared to uninfected mice (Fig. 3G and fig. S6). In addition, the expression of the leukocyte adhesion molecules, *Icam* and *Vcam*, were significantly elevated in x31 infected WT mice compared to both uninfected WT and x31 infected TLR7^−/−^ mice (fig. S6).

**Fig. 4.**
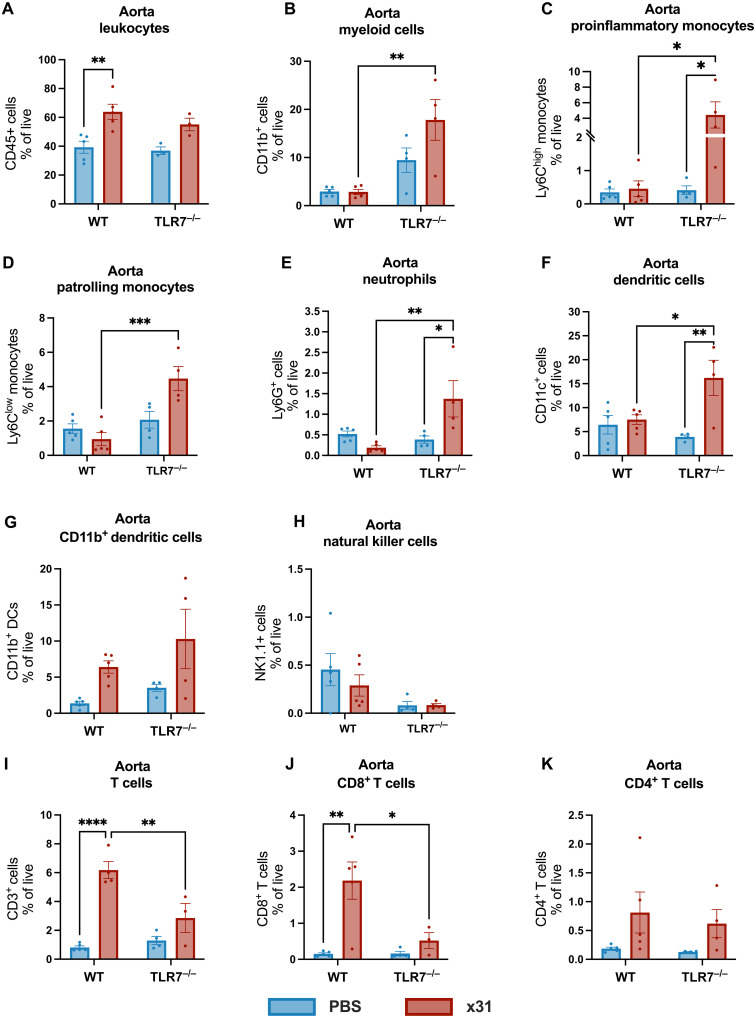
TLR7 promotes T cell recruitment to the aorta during gestational IAV infection. The aorta of pregnant WT or TLR7^−/−^ dams infected with Hk-X31 (x31; 10^4^ PFU) or mock infected with PBS was taken at 6 dpi, and single-cell suspensions were generated to investigate the changes in (**A**) leukocytes, (**B**) myeloid cells, (**C**) pro-inflammatory monocytes, (**D**) patrolling monocytes, (**E**) neutrophils, (**F**) DCs, (**G**) CD11b^+^ DCs, (**H**) NK cells, (**I**) T cells, (**J**) CD4^+^ T cells, and (**K**) CD8^+^ T cells by flow cytometry. Data are presented as the proportion of live cells expressing the specific marker. Statistical analysis was determined via two-way ANOVA with Tukey’s post hoc test (*P ≤ 0.05; **P ≤ 0.01; ***P ≤ 0.001; ****P ≤ 0.0001). All data are presented as means ± SEM. *n* = 4 to 5 per group.

Given that one of the hallmarks of the “vascular storm” caused by gestational IAV infection is excessive aortic immune cell infiltration (*13*), we next investigated the role of TLR7 in regulating vascular immune cell recruitment. Although the total frequency of CD45^+^ leukocytes infiltrating the aorta (Fig. 4A) was similar between infected pregnant WT and TLR7^−/−^ mice, we observed distinct differences in the immune cell populations recruited to the aorta. Specifically, infected TLR7^−/−^ mice showed significantly higher frequencies of myeloid cells, Ly6C^high^ and Ly6C^low^ monocytes, neutrophils, and DCs when compared to WT mice (Fig. 4, B to F), with no significant differences in the proportions of CD11b^+^ DCs or NK cells (Fig. 4, G and H). Conversely, infiltration of CD3^+^ T cells, particularly CD8^+^ T cells into the aorta, was significantly reduced in infected TLR7^−/−^ mice compared to WT mice (Fig. 4, I and J), whereas no significant differences in the infiltration of CD4^+^ T cells was observed (Fig. 4K). These findings suggest that TLR7 plays a crucial role in orchestrating the immune response in the aorta during gestational IAV infection, particularly by modulating the recruitment of myeloid cells and lymphocytes.

### TLR7 regulates inflammation at the maternofetal interface during IAV infection

**Fig. 5.**
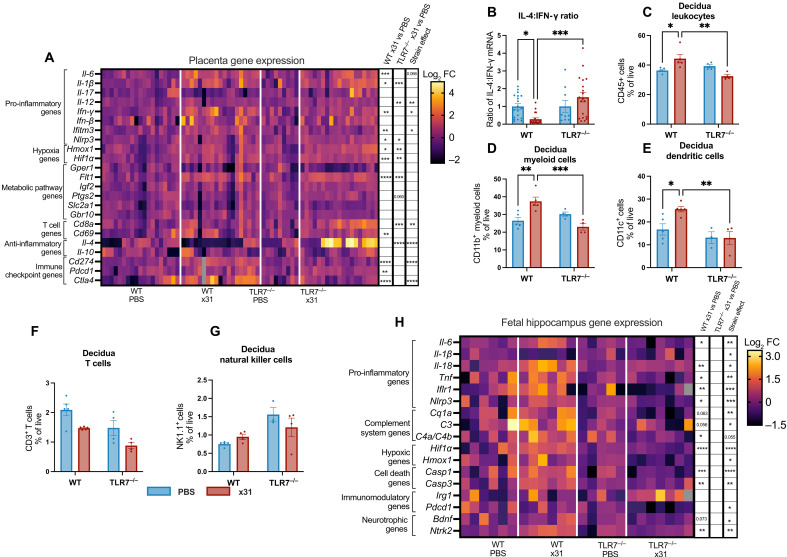
TLR7 deletion restricts T_H_1-skewed immunity in the placenta and protects against fetal brain neuroinflammation during gestational IAV infection. Pregnant WT or TLR7^−/−^ dams infected with Hk-X31 (x31; 10^4^ PFU) or mock infected with PBS at 6 dpi. (**A**) Three placentae per litter were harvested to assess inflammatory gene expression via qPCR. Gene expression was normalized to the expression of *Ywaz* and expressed as the binary logarithm (log_2_) of the fold change of the geometric mean of the PBS controls. Gray box indicates the sample was missing from this gene analysis. (**B**) T_H_2 to T_H_1 cytokine skewing was assessed as the ratio of *Il-4* to *Ifn-*γ expression. (**C** to **G**) To assess the infiltration of immune cells to the decidua, single-cell suspensions were generated from three decidua isolated per litter and analyzed for cell phenotype by flow cytometry and expressed a proportion of live cells. (**H**) Gene expression was assessed in the hippocampus of E18 offspring of infected or mock-infected dams. qPCR analysis of pro-inflammatory cytokine, complement system, hypoxic, cell death, immunomodulatory, and neurotrophic genes was normalized to the expression of *Gapdh* and expressed as the binary logarithm (log_2_) of the fold change of the geometric mean of the PBS controls. Gray box indicates the sample was missing from this gene analysis. Statistical analysis was determined via two-way ANOVA with Tukey’s post hoc test for multiple comparisons. Strain effect denotes statistical significance between infected genotypes (x31 WT versus x31 TLR7^−/−^) [(A) and (H)] (*P ≤ 0.05; **P ≤ 0.01; ***P ≤ 0.001; ****P ≤ 0.0001). All data are presented as means ± SEM. [(A) and (B)] *n* = 9 to 19, [(C) to (G)] *n* = 4 to 5, and (H) *n* = 4 to 7 per group.

Gestational IAV infection has been evidenced to result in offspring that are small for gestational age (*26*). In our study, offspring from infected TLR7^−/−^ mice were spared from this complication, prompting us to investigate whether this improvement in fetal growth was associated with gene transcriptional alterations in inflammation, hypoxia, and the metabolic environment at the placenta [Fig. 5A (heatmap) and fig. S7 (individual graphs)]. Gestational IAV infection was associated with increased placental gene expression of *Il-1*β, *Nlrp3*, *Hmox1*, *Hif1a*, and *Flt1* in both WT and TLR7^−/−^ mice. In contrast, placental *Il-6*, *Ifn-*γ, *Ifitm3*, *Cd69*, *Cd274*, *Pdcd1*, and *Ctla4* were significantly up-regulated in WT mice following x31 infection but not in infected TLR7^−/−^ mice, whereas placental *Il-12a*, *Cd8a*, and *Il-4* mRNA were significantly increased after IAV infection in TLR7^−/−^ mice but not in infected WT mice. Assessment of the T helper 1 (T_H_1)/T_H_2 cytokine profile using the ratio *Il-4* to *Ifn-*γ revealed a T_H_1-skewed response in WT mice but not in TLR7^−/−^ mice following x31 infection (Fig. 5B). Together, these data indicate that, in the absence of TLR7, the placental environment maintains a T_H_2-skewed profile and avoids a shift toward T_H_1 mediated immunity during maternal IAV infection, the latter of which has been previously shown to negatively affect pregnancy outcomes (*27*, *28*). In response to infection, WT mice displayed increased CD45^+^ leukocyte infiltration into the decidua, the maternal interface of the placenta (Fig. 5C). This was largely driven by increased numbers of myeloid cells (CD11b^+^) and DCs (CD11c^+^) compared to infected TLR7^−/−^ mice (Fig. 5, D to F), whereas no significant difference was observed in the frequency of T cells (CD3^+^) or NK cells (NK1.1^+^; Fig. 5G and fig. S8, A to E). In contrast, gestational IAV infection of TLR7^−/−^ mice revealed no significant changes to the decidual immune cell population. In addition, we show that the infiltration of immune cells at the decidua does not extend into the fetal compartment of the placenta nor is viral RNA detectable in the placentae (fig. S8, F to K).

### TLR7 deletion during gestational IAV infection rescues fetal neuroinflammation

Maternal IAV infection is associated with neurodevelopmental disorders in the offspring, including schizophrenia (*29*). We therefore sought to assess the impact of both IAV infection and TLR7 on the inflammatory and cellular environment within the hippocampus as this region shows the greatest structural and functional changes in schizophrenia (*30*). Consistent with our previous findings (*31*), we find that x31 infection of WT pregnant mice resulted in pronounced expression of neuroinflammation and cell death markers in the E18 fetal hippocampus [Fig. 5H (heatmap) and fig. S9 (individual graphs)]. x31 WT offspring displayed significantly increased levels of the hypoxic marker, *Hif1α*, compared to x31 TLR7^−/−^ offspring. Similar increases were observed with pro-inflammatory cytokines, with only x31 WT offspring showing increased levels of *Il-6*, *Tnf*, and *Il-18* gene expression. Genes involved in neurotrophin signaling, brain-derived neurotrophic factor (*Bdnf*) and its receptor neurotrophic receptor tyrosine kinase 2 (*Nrtk2*), were also found to be significantly higher in x31 WT offspring compared to x31 TLR7^−/−^ offspring.

x31 WT offspring also display significantly elevated markers of cellular death, with increases in markers of both apoptosis (*Casp3*) and pyroptosis (*Casp1*). x31 WT offspring also had increased levels of the inflammasome marker, *Nlrp3* compared to x31 TLR7^−/−^ offspring. Maternal IAV infection was also found to be associated with increased fetal brain expression of complement system genes. This was consistent with previous clinical data that showed elevated expression of complement system components within the brain of patients with schizophrenia (*32*). The levels of complement genes *C1qa*, *C3*, and *C4a/4b* were significantly higher in x31 WT offspring and contrasted with x31 TLR7^−/−^ offspring, which displayed no IAV-induced increase in complement. Together, this indicated that TLR7 orchestrates offspring neuroinflammation in utero during maternal IAV infection.

## DISCUSSION

Here, we present evidence for an important role for TLR7 in mediating maternal and fetal morbidity during gestational IAV infection. We show that the TLR7 signaling pathway leads to increased systemic disease, contributing to a “vascular storm” that results in inflammation that can cause pulmonary and cardiovascular pathology. Furthermore, the presence of viral RNA in the maternal cardiovascular system in WT mice was associated with in utero growth retardation and fetal neuroinflammation. Given the generally low propensity for vertical IAV transmission (*33*, *34*), we hypothesize that fetal complications arising from gestational IAV infection are the result of aberrant maternal immune activation mediated by TLR7. Our results highlight TLR7 as a central mediator of both maternal and fetal pathophysiology, offering a potential therapeutic target to mitigate pregnancy-related complications during viral infections.

Pregnancy is a unique physiological state that influences maternal immune responses to protect rejection of the semiallogenic fetus; however, this modulation of maternal immunity often results in altered susceptibility to infections and immune-mediated diseases. Previous studies have shown that gestational IAV infection is associated with increased systemic disease compared to infected, nonpregnant controls (*35*, *36*). In particular, we have previously shown increased extra-pulmonary viral RNA detection, including a notable 10-fold rise in aortic viral RNA relative to nonpregnant controls (*13*). One factor that could contribute to this heightened viral spread is the regulation of inflammation in the lungs, where viral replication and immune activation can influence the extent of extra-pulmonary viral RNA. Our current study identifies a positive correlation between aortic viral titers and *Osm* gene expression in WT lungs, suggesting that *Osm* may play a key role in this process. Osm, an Il-6 family cytokine, promotes epithelial and endothelial barrier disruption, through STAT3 signaling, which ultimately down-regulates the expression of tight junction proteins (*37*, *38*). In the context of influenza, the expression of Osm is correlated to a decrease in the tight junction proteins, Zona Occludins-1, Occludin, and Cadherin-1 (*25*). Given the positive correlation between lung *Osm* levels and aortic viral RNA, Osm could be useful as a potential biomarker for assessing the risk of IAV-dependent complications in pregnant women. Future studies should investigate Osm in nasal brushings or sputum samples from infected pregnant women and explore whether these levels correlate with pregnancy complications. Mechanistically, further studies are required to understand the physiological role of Osm during pregnancy and its contribution to increased susceptibility to viral dissemination. This could assist in developing new antiviral therapies that target Osm to reduce IAV morbidity.

Previous research has established that *Osm* expression in the lungs is regulated through IFN-I signaling via the IFN-α/β receptor (IFNAR) (*39*). In this study, we demonstrate that this *Osm*-viral titer correlation is mediated by a TLR7-dependent pathway, linking TLR7 activation, IFN-I signaling, and *Osm* expression. Because TLR7 activation is one of the primary drivers of IFN-I production during viral infections, the absence of TLR7 in mice leads to significantly lower *Ifn-*β levels compared to WT animals, supporting the importance of TLR7 in initiating the antiviral immune response. Although IFN-I signaling plays a critical role in limiting viral replication and enhancing immune defenses, *Osm*—known to disrupt epithelial tight junctions—has been shown to increase lung inflammation and immune cell infiltration during IAV infection (*25*, *40*). This suggests that *Osm* could contribute to the lung damage associated with viral infections, particularly in the context of pregnancy. In our model, we propose that reduced IFN-I signaling and *Osm* expression in TLR7^−/−^ mice may serve a protective role by preserving the integrity of the epithelial barrier in the lungs. Supporting this hypothesis, we observe that TLR7^−/−^ mice display less edema and alveolar and peribronchiolar inflammation compared to WT mice, suggesting that TLR7 deficiency mitigates IAV-induced lung pathology. This finding implies that Osm and IFN-I signaling may be tightly interconnected in regulating the balance between effective antiviral immunity and detrimental lung inflammation.

IFN-II is another antiviral cytokine that acts in concert with IFN-I to limit viral replication and the mounting of an adaptive immune response. In contrast to the IFN-I responses in WT mice, infected WT pregnant mice failed to induce an IFN-II response, whereas TLR7^−/−^ pregnant mice had a robust IFN-II response to IAV. In this study, we did not observe significant differences in the number of NK and CD8^+^ T cells in the lungs of infected pregnant WT and TLR7^−/−^ mice, both of which are major sources of IFN-II. This suggests that another cell type may be responsible for the increased pulmonary IFN-II in TLR7^−/−^ mice or perhaps TLR7 is modulating NK and CD8^+^ T cell effector functions in pregnancy. A previous study has shown that migratory neutrophils produce IFN-II early during *Streptococcus pneumoniae* infection, which is critical for bacterial clearance (*41*). This neutrophilic-driven IFN-II response was not dependent on IL-12 (which is also involved in T_H_1 differentiation) and the activation of TLR2 and TLR4 (*42*). In this study, we show increased neutrophil infiltration into the lungs and aorta of infected pregnant TLR7^−/−^ mice. Neutrophil effector functions play a dual role at the maternal-fetal interface, by facilitating physiological tissue remodeling during pregnancy while also acting as the cellular drivers of excessive inflammation and adverse pregnancy outcomes during maternal infection (*43*). Whether TLR7 is also dispensable in neutrophilic IFN-II responses during IAV infection and its consequences on the maternal cardiorespiratory system and maternal-fetal interface requires further investigation. However, there is evidence to show that TLR7 activation in neutrophils shifts their function from a phagocytic phenotype toward the more pro-inflammatory and tissue damaging NETosis (*41*). Together, we propose that blocking TLR7 during IAV infection may mitigate pulmonary pathology by suppressing NETosis-mediated tissue damage while still maintaining effective viral clearance through IFN-II production.

Although gene expression profiling does not capture posttranslational modifications or protein activity, it provides a powerful and holistic overview of the transcriptional programs underpinning inflammation, antiviral signaling, and immune regulation. Our findings therefore offer strong mechanistic insight into TLR7-dependent pathways during gestational IAV infection; nevertheless, future studies using proteomic and functional assays will be important to validate and extend these transcriptional observations.

We have previously shown that IAV infection during pregnancy drives maternal endothelial dysfunction, as defined as the loss of vasodilatory response to ACh, and is associated with increased TLR7 expression (*13*, *44*). Activation of TLR7 with agonists like R837 and CLO97 induces pregnancy-dependent increases in systolic blood pressure and aortic endothelial dysfunction in mice (*44*). In our study, pregnant TLR7^−/−^ mice revealed impaired basal responsiveness to ACh compared to pregnant WT mice. Moreover, in contrast to pregnant WT mice, IAV infection did not exacerbate endothelial dysfunction in the TLR7^−/−^ mice. Despite the increased cardiovascular demands of pregnancy, WT-infected dams displayed reduced heart rate and systolic pressure, consistent with infection-induced vascular dysfunction. Although we drew mechanistic parallels with hypertensive disorders of pregnancy (shared endothelial injury and inflammatory vasculopathy), the net phenotype in our model is hypotension, reinforcing that TLR7-driven inflammation impairs vascular tone rather than elevating blood pressure. Therefore, we propose that TLR7 plays a more complex role in regulating vascular tone beyond its classical immunological functions. Others have reported TLR7-mediated regulation in airway constriction implicating TLR7 expressed by airway neurons as a source of nitric oxide (*45*). Further investigation is required to determine whether TLR7 also regulates nitric oxide production in the aorta. These findings suggest that TLR7 drives vascular pathology through immune-mediated mechanisms in addition to reducing aortic viral burden. The mechanistic link between TLR7 signaling and endothelial dysfunction likely extends beyond viral load. Our data show that TLR7 activation is associated with increased vascular expression of cytokines (e.g., *Osm* and *Il-1*β) and adhesion molecules (*Icam* and *Vcam*), which may promote T cell adhesion and transmigration into the aortic wall. In addition, TLR7 has been reported in nonimmune cells, including vascular endothelium and neurons, where its activation can influence nitric oxide production and smooth muscle contractility, suggesting a direct role in modulating vascular tone independent of viral titers. Last, the observed accumulation of CD8^+^ T cells in the aorta of WT dams, but not TLR7^−/−^ dams, raises the possibility of a feed-forward loop in which TLR7-driven immune activation promotes T cell recruitment, thereby amplifying endothelial injury and vascular dysfunction. Future studies using conditional knockouts and endothelial functional assays will be important to dissect these pathways in greater detail.

Although pregnant TLR7^−/−^ mice showed significantly reduced baseline responsiveness to ACh compared to pregnant WT mice, measures of maternal cardiac function (pulse rate and systolic blood pressure) remained unchanged in response to infection. Moreover, their offspring remained protected against IAV-induced fetal growth restriction and neuroinflammation in utero. These observations suggest that preventing viral dissemination and/or TLR7 signaling during gestational IAV infection can lead to improved pulse rate and systolic blood pressure, which, in turn, may improve placental perfusion and promote better fetal growth and down-regulate neuroinflammatory signatures associated with neurodevelopmental risk, regardless of aortic function. Although pregnancy is a high-risk group for influenza-triggered complications, the effects of infection on the maternal cardiovascular system have been insufficiently studied in pregnant women. Furthermore, given that evidence of endothelial-dependent vascular dysfunction has been associated with poor fetal and maternal outcomes in other conditions, such as preeclampsia, fetal growth restriction and gestational diabetes (*46*–*48*), the monitoring of maternal cardiac output during infection could provide valuable insight into the risk of fetal complications resulting from gestational respiratory infections.

Studies have shown T cells play an important role in regulating vascular tone (*49*) and endothelial dysfunction in hypertensive disorders (*50*, *51*). We have previously shown that gestational IAV infection is associated with a significant increase in aortic T cell accumulation in pregnant mice and that this influx of aortic T cells persists postpartum and long after viral clearance (*35*). Here, IAV infection in pregnant WT mice induced a robust aortic T cell response, which was associated with endothelial dysfunction. In contrast, although CD45^+^ leukocyte recruitment to the aorta was similar between infected pregnant TLR7^−/−^ and WT mice, aortic T cell numbers in response to infection were reduced in pregnant TLR7^−/−^ mice. Together, these observations highlight the need for further studies to explore the role of TLR7 in aortic T cells and their influence on vascular tone during viral infections and under baseline conditions.

Studies have shown that pregnant women have T cells that are hyperresponsive to IAV compared to nonpregnant women (*52*). In this study, reduced aortic viral RNA was associated with both reduced aortic T cell infiltration and overall vascular inflammation in IAV-infected pregnant TLR7^−/−^ mice. In pregnant infected TLR7^−/−^ mice, reduced vascular inflammation was associated with a lack of fetal neuroinflammation despite equivalent maternal pulmonary inflammation between TLR7^−/−^ and WT mice. Thus, it could be argued that the systemic “vascular storm” exacerbated by excessive aortic T cell recruitment and activation in response to gestational IAV infection may skew a healthy immune response into a destructive one, resulting in aberrant fetal growth and neuroinflammation. The direct effects of TLR7 activation on T cell function has been revealed to be complex, with one study reporting TLR7 signaling in T cells inhibiting pro-inflammatory T_H_1 and T_H_17 differentiation (*53*), whereas others have shown that excessive TLR7 signaling in T cells promotes T_H_1 and T_H_17 differentiation (*54*). In addition to the intrinsic effects of TLR7 signaling on T cell function, others have shown indirect effects of TLR7 in modifying T cell responses. TLR7 activation of DCs has been demonstrated to promote T_H_17 differentiation and IL-17 production (*55*, *56*). It is currently unclear in our study to what extent does TLR7 modulates T cell activation during IAV infection in pregnancy, and this warrants further investigation. Together, we propose that fetal neuroinflammation caused by gestational IAV is tied to the establishment of the “vascular storm,” a process that seems to be primarily dependent on TLR7, rather than the magnitude of maternal pulmonary inflammation caused by the virus.

Dysfunction and inflammation at the maternal-fetal interface (including the decidua and placenta) underlies many common pregnancy and fetal complications (*57*). Although IAV is not vertically transmitted to the fetus, maternal IAV infection is associated with placental inflammation, tissue damage, and necrosis (*58*). In this study, decidual DCs, NK cells, and T cells were significantly reduced in IAV-infected TLR7^−/−^ pregnant mice, indicating that TLR7 promotes IAV-induced inflammation at the maternal-fetal interface. We propose that IAV-induced inflammation at the maternal-fetal interface is the result of the upstream maternal pulmonary and cardiovascular inflammatory environment driven by TLR7. Here, TLR7 did not influence pulmonary viral titers, suggesting that TLR7 is dispensable for effective viral control in the lungs, whereas loss of TLR7 provided protection against the development of the “vascular storm” in infected pregnant mice. We have previously shown that targeting the “vascular storm” with low-dose aspirin, which reduces vascular inflammation and dysfunction with minimal effects on pulmonary inflammation and viral clearance, can ameliorate placental and fetal complications associated with gestational IAV infection (*59*). Together, we propose that the fetal complications arising from gestational IAV infection are driven by the development of the “vascular storm,” which leads to maternal vascular dysfunction and therefore suboptimal blood flow to the placenta, resulting in neuroinflammation and potential hypoxia-induced brain injury. Nevertheless, this study demonstrates the systemic damaging effects of IAV infection during pregnancy mediated by TLR7 outside of the respiratory tract, causing major disruptions in the maternal cardiovascular system and the placental environment and triggering fetal neuroinflammation.

This study has several limitations that should be acknowledged. First, the effect of fetal sex was only assessed in the placenta and not the fetal hippocampus, and given known sex-specific differences in immune, placental, and neurodevelopmental trajectories, future work should incorporate sex determination to identify whether TLR7-dependent effects differ between male and female offspring. Second, our analyses focused on transcriptional signatures and immune cell profiling; protein expression, posttranslational modifications, and functional assays were not assessed. Because lungs were not perfused before isolation, minor blood contamination cannot be excluded and may have modestly influenced the quantification of patrolling Ly6C^low^ monocytes. Third, our findings that TLR7 promotes CD4^+^ and CD8^+^ T cell recruitment to the maternal aorta suggest a previously underappreciated link between antiviral sensing, vascular immune infiltration, and endothelial dysfunction. Although we did not distinguish T cell subsets in this study, future work using expanded panels will be essential to determine how regulatory versus pro-inflammatory T cell subsets, including T_H_17, regulatory T cells, and γδ T cells, contribute to TLR7-driven vascular pathology during gestational influenza. Fourth, we did not perform gene expressional profiling on specific placenta zones (junctional versus labyrinth), which may differentially contribute to maternal-fetal immune regulation. Fifth, although we identified robust neuroinflammatory signatures in the fetal hippocampus, we did not evaluate anatomical changes in hippocampal specific subregions (CA1, CA3, and DG) or behavioral outcomes postnatally, which will be essential to establish links between TLR7 and long-term neurodevelopment. Sixth, the smaller number of dams in the uninfected TLR7^−/−^ group reflects breeding constraints rather than study design. Limited animal availability necessitated prioritization of this genotype within the infected cohort to maintain statistical power for assessing infection-driven effects. Last, although our cardiovascular findings highlight TLR7 as a regulator of vascular dysfunction, detailed assessments of vasoconstrictor sensitivity and maternal cardiac output were beyond the scope of this study. Future studies integrating these approaches will be critical to build a comprehensive mechanistic framework. Despite these limitations, our findings uncover a previously unrecognized role for TLR7 as a master regulator of maternal-fetal immune and vascular pathology during gestational influenza, providing a strong conceptual and translational foundation for targeting TLR7 to prevent infection-associated pregnancy complications.

In summary, this study provides insight into a previously unidentified mechanism into how gestational influenza drives maternal and fetal pathology, identifying TLR7 as a central regulator of immune activation, vascular dysfunction, placental inflammation, and fetal neuroinflammation. Our findings demonstrate that TLR7-dependent systemic inflammation and endothelial dysfunction occur in the context of low-level extra-pulmonary viral RNA, rather than being strictly proportional to viral burden. Together, these changes converge to promote decidual and placental inflammation, impaired fetal growth, and neuroinflammatory signatures associated with neurodevelopmental risk. These data position TLR7 as a promising therapeutic target for mitigating maternal and fetal morbidity during severe influenza in pregnancy. Preclinical studies have shown that activation of placental TLR7 signaling reduces expression of placental efflux transporters, which play a crucial role in protecting the fetus from xenobiotic accumulation (*60*). Clinically, endosomal TLR7/9 antagonism with hydroxychloroquine has an established record of controlled use in pregnancy, whereas selective TLR7/8 inhibitors (e.g., enpatoran) are investigational and lack obstetric safety data; thus, any translational pathway will require drug-specific evaluation despite encouraging preclinical rationale. Moreover, although direct evidence linking TLR7 variants or expression to severe gestational influenza is currently lacking, TLR7 is expressed at the maternal-fetal interface (*44*) and exhibits female-biased, variant-dependent IFN responses (*61*), underscoring the need for prospective obstetric studies integrating TLR7 genotyping, expression profiling, and clinical outcomes.

## MATERIALS AND METHODS

### Mouse models

All animal experiments were conducted with approval from the Royal Melbourne Institute of Technology (RMIT) University Animal Ethics Committee (study approval number 24336) and in compliance with the National Health and Medical Research Council of Australia “Australian code for the care and use of animals for scientific purposes” guidelines (*62*).

Time-mated, pregnant WT (C57BL/6JOzarc) mice were purchased from OzgeneARC (Australia), and homozygous TLR7^−/−^ (fig. S10; B6.129S1-Tlr7tm1Flv/J) were obtained from the Jackson Laboratory (catalog no. 008380) and time-mated at the RMIT University Animal Facility (Bundoora, Australia). Nulliparous, age-matched (8 to 12 weeks old) mice were time mated before being transferred onto the experimental protocol at E11.5 of the pregnancy.

### Virus

The mouse-adapted pandemic (H3N2) IAV strain, Influenza A/HKx31 (x31), was kindly provided by P. Reading (The Peter Doherty Institute for Infection and Immunity, University of Melbourne). Working stocks of x31 were propagated under standard conditions using embryonated day 10 chicken egg cells, and progeny viruses were collected from the supernatant and quantified using standard plaque assay procedures in Madin-Darby canine kidney cells (*63*, *64*). Aliquots of x31 were generated at a concentration of 9.6 × 10^7^ PFU/ml and stored at −80°C until required for use.

### Infections and tissue collection

Mice were acclimatized for 24 hours before infection. Pregnant dams were infected at E12.5 of gestation with IAV by intranasal inoculation with 10^4^ PFU in 35 μl of sterile phosphate-buffered saline (PBS), or an equivalent volume of PBS was used for uninfected control mice; dams were anesthetized by isoflurane inhalation (3% in oxygen) for 6 min. Mice were monitored daily, and gross body weights were recorded. Dams were euthanized 6 dpi (E18.5) via intraperitoneal injection of sodium pentobarbital (240 mg/kg) in a volume of 200 μl. Once animals were deeply anesthetized—as determined by a lack of response to stimuli such as the toe-pinch reflex or whisker reflex—cardiac puncture was performed to collect whole blood. Whole blood (0.5 to 1 ml) was collected and transferred into EDTA-lined 1-ml K3 EDTA MiniCollect tubes (Greiner Bio-One). Samples were inverted several times to ensure contact of all blood with the EDTA surface to prevent coagulation. Samples were then centrifuged at 10,000*g* for 10 min at 4°C, and plasma was collected and transferred to a 1.5-ml Eppendorf tube. Maternal lungs, heart, aorta, and BALF were also collected, and tissue masses were recorded. Fetal and placental mass were recorded for each viable pup, and three placenta and three fetal brains (per litter) were randomly selected; the hippocampus (containing the CA1, CA3, and DG subregions) of each fetal brain was isolated, as previously described (*65*), under stereology microscopy and pooled together on the basis of litter for downstream quantitative polymerase chain reaction (qPCR) analysis. All physiological measurements and tissue collections were performed in the morning between 9:00 a.m. and 12:00 p.m. to minimize the influence of circadian variation. A total of 32 WT and 37 TLR7^−/−^ dams were used in this study. At least two independent inoculations were performed for each genotype for all experiments.

### Airway inflammation analysis

The BALF was collected to assess pulmonary inflammation. Briefly, lungs were lavaged with 300 to 400 μl of cold PBS repeatedly for a total volume of 1.2 ml. The total number of viable cells within the BALF was by staining with 10 μl of Acridine Orange (Thermo Fisher Scientific). Infected (x31) mice had BALF samples diluted to 1:10, and mock-infected mice (PBS) were diluted to 1:2 using PBS. The diluted and Acridine Orange stained samples were then transferred to a hemocytometer to quantify the total number of live cells via fluorescence microscopy, and the total number of viable cells was recorded as live cells/ml of BALF.

To determine the immune cell profile of the airways following IAV infection, differential analysis was conducted on cells isolated from the BALF. Briefly, BALF cells were centrifuged at 400*g* for 5 min at 4°C. The supernatant was then discarded. Cells were then resuspended in sterile PBS at a final concentration of 2.5 × 10^4^ cells/ml. An aliquot of 5 × 10^4^ cells (200 μl) was then transferred onto a Superfrost plus microscope slide (Thermo Fisher Scientific) via centrifugation in a Shandon Cytospin 3 Centrifuge (Thermo Fisher Scientific) for 5 min at 112*g*. Differential staining was then performed using the Shandon KwikDiff Staining kit (Thermo Fisher Scientific) as per the manufacturer’s protocol. Analysis of BALF cell composition involved the quantification of immune cells populations including monocytes, lymphocytes, neutrophils, and eosinophils based on standard morphological criteria. A total of 500 cells were counted over five randomly selected regions across the slide, and the proportion of the individual population, as well as the absolute number of cells (based on total live cell counts), was calculated.

### Lung histology

To assess histopathology, the left lung of dams was collected and fixed in 10% neutral buffer formalin for a minimum of 24 hours before being dehydrated with ethanol and embedded in paraffin wax blocks. Five-micrometer-thick sections of the lung were mounted onto glass slides and stained using hematoxylin and eosin (H&E) to assess for morphological changes following infection. Briefly, tissue was deparaffinized by placing slides into a dry oven for 10 min at 60°C and submerging slides in xylene. Tissue rehydration was performed using graded ethanol washes of 1 min each (100% twice and 90, 70, and 50%) and then rinsed in Milli-Q H_2_O. Slides were then immersed in hematoxylin and “Scott’s Tap Water” (0.083 M MgSO_4_ and 0.23 M NaHCO_3_), followed by counterstaining with eosin for 90 s and rinsing with Milli-Q H_2_O. Following staining, slides were dehydrated with graded ethanol washes for 1 min (50, 70, and 90% and 100% twice). Slides were then mounted with DPX Mounting media. Tissues were visualized using an Olympus VS120 slide scanner (Olympus Life Sciences). Whole-tissue sections were scanned and imaged using a ×40 magnification lens under bright-field conditions. Structural changes within the lungs following influenza infection were assessed using three parameters of lung pathology—alveolitis, peribronchiolar inflammation, and inflammatory cell infiltration—these markers of lung pathology were rated on the basis of visual examination of tissue sections, where two blinded independent assessors provided a rating of pathological severity using a scale of 0 to 5, where 0 indicates no presence of tissue abnormality and 5 indicates the most severe pathological score. The average score for each parameter was taken for the respective tissue sample, and once all tissues were assessed, sample IDs were unblinded and group analysis was conducted to determine variance across infection and strains.

### Flow cytometry

Flow cytometry was conducted to assess the immune cell composition across various anatomical sites within mice during the acute phase (6 dpi) of IAV infection. Tissues were collected from a separate cohort of dams that were allocated specifically for flow cytometry analysis. Dam (aorta, lung, and heart) and fetal (placenta and decidua) tissues were isolated from animals at time of euthanasia. Tissues were placed into 1.5-ml Eppendorf tubes containing 1 ml of PBS and placed on ice to maintain cell viability during collection. Maternal aorta was collected and encompassed the aortic arch through to the iliac bifurcation with abdominal perivascular, and para-aortic lymph nodes were removed. From each litter, three placentae were collected and the decidua of each placenta was carefully removed and placed into separate Eppendorf tubes. All tissues underwent mechanical digestion with scissors before being placed into an appropriate enzymatic digestion buffer based on tissue composition. Lung, placenta, and decidua were digested in 4 ml of Liberase TM (50 μg/ml; diluted in Hanks’ balanced salt solution) (Sigma-Aldrich) using a thermomixer set to 750 rpm and at 37°C for either 45 min (lungs) or 25 min (placenta and decidua). Following enzymatic digestion, lung tissues were further homogenized using 18-gauge, followed by 21-gauge, sterile needles attached to 5-ml syringes. Aortic sections underwent enzymatic digestion for 1 hour at 37°C with gentle agitation using a buffer composed of 125 U collagenase type XI, 225 U hyaluronidase, and 225 U collagenase type I-S in PBS with Ca^2+^ and Mg^2+^. Heart tissue was likewise enzymatically digested in a buffer containing 200 U collagenase type I-S made up to 1 ml in RPMI 1640 for 25 min using a thermomixer set to 750 rpm and at 37°C. Samples were then filtered through 40-μm (lung, heart, decidua, and placenta) and 70-μm (aorta) filters and pelleted by centrifugation at 400*g* for 5 min at 4°C. Red blood cell (RBC) lysis buffer (155 mM NH_4_Cl, 9.98 mM KHCO_3_, and 0.1 mM EDTA in H_2_O) was added to all samples for 2 to 3 min to lyse any contaminating RBCs. Samples were resuspended in flow cytometry [fluorescence-activated cell sorting (FACS)] buffer (2.5% fetal bovine serum in PBS) for antibody staining. Single-cell suspensions of each tissue were transferred in a volume of 200 μl to 96-well plates. All samples were stained to assess viability with LIVE/DEAD Aqua for 15 min at 4°C. Staining of surface markers was conducted by incubating cells with antibody cocktails diluted in FACS buffer for 25 min at 4°C. Cell surface antibodies used were (BioLegend) as follows: CD45-AF700 (clone 30-F11), CD45-BV650 (30-F11), CD3-APC (145-2C11), CD4-BV605 (RM4-5), CD8-PE-Cy7 (53-5.8), CD11b-BV421 (m1/70), Ly6C-FITC (HK1.4), Ly6G-APC-Cy7 (1A8), NK1.1-BV605 (PK136), CD11c-PE-Cy7 (N418), and CD103-AF700 (2E7). Single stain controls were generated using splenocytes from WT mice. To determine the appropriate staining pattern and intensity for viable cells, ~1 × 10^6^ splenocytes were incubated at 56°C for 10 min to induce cell death. Following immunostaining, cells were fixed using the BD Fixation Buffer, per the manufacturer’s protocol, and then washed and resuspended in 200 μl of FACS buffer and stored overnight at 4°C. Samples were analyzed for fluorescence signal using the FACSAria Fusion flow cytometry system using the associated FACSDiva software. A minimum of 1 × 10^6^ events were acquired for lung, heart, and placental tissues, and a minimum of 6 × 10^5^ were acquired for decidua and aorta tissue samples. Following collection of data, samples were analyzed for cellular populations using the FlowJo software package. Populations of cells were expressed as a proportion of live cells, unless stated otherwise. The gating strategy for population analysis for both cocktails and representative histogram of viability staining is shown in fig. S2.

### RNA extraction and cDNA synthesis from dam and fetal tissues

RNA was isolated from lung, heart, and aortas collected from dams euthanized at 6 dpi and E18.5 fetal brain and placentae. Snap-frozen heart, lung, and whole placentae were ground to a fine powder using a stone mortar and pestle cooled with liquid nitrogen. Approximately 25 μg of tissue was transferred into a 1.5-ml Eppendorf tube containing 350 μl of β-mercaptoethanol diluted 1:100 in RLT buffer (QIAGEN; catalog no. 79216), before manual homogenization by passing the suspension through a 21-gauge needle and syringe five times. The β-mercaptoethanol–tissue mixture was then centrifuged for 5 min at 10,000*g* at 4°C to clarify the supernatant. The supernatant was then mixed with an equal volume of 70% ethanol.

Lipid-rich tissue, such as the fetal hippocampus (incorporating CA1, CA3, and DG subregions) and dam’s aorta [with surrounding intact perivascular adipose tissue (PVAT)], was homogenized using a TRIzol-based method. Briefly, 1 ml of TRI Reagent (Thermo Fisher Scientific; Massachusetts, USA), and 5-mm stainless steel beads (QIAGEN; Walden, Germany) were added to each 2-ml safe-lock Eppendorf tube containing frozen tissue. The tissue was then bead homogenized twice using the TissueLyser LT (QIAGEN; Hilden, Germany) for 5 min at maximum speed (50 Hz). Samples were placed on ice to cool down between homogenization steps. The homogenate was then combined with 200 μl of chloroform before being centrifuged at 10,000*g* for 15 min at 4°C. The separated upper aqueous phase was then carefully removed and transferred into a fresh 1.5-ml Eppendorf tube containing 600 μl of 70% ethanol. The resulting tissue homogenate–ethanol solutions were then transferred to a QIAGEN RNeasy spin column and RNA was isolated per the manufacturer’s protocol, with the RNA eluted in 30 μl of RNase-free water. The isolated RNA then underwent cDNA synthesis using the High-Capacity cDNA Reverse Transcription kit as per the manufacturer’s protocol. Either 1 or 2 μg of total RNA was added to each reaction for a final reaction volume of 20 μl.

### Quantification of virus in dam tissues

Quantification of viral RNA was performed via reverse transcription qPCR (RT-qPCR) using synthesized cDNA from dam tissues using a previously published custom designed primer set for the segment-3 polymerase (PA) gene of IAV (*66*). Uninfected tissue controls for both genotypes were used as negative controls for RT-qPCR experiments. Viral RNA levels were normalized to the expression of *RPS18* to account for variability, using the Delta-CT method (*67*), and reported as a relative ratio (fold change) respective to mean of uninfected genotype control group. Delta-CT calculations and raw CT values are displayed in fig. S10 (D to I).

### Quantification of mRNA expression by RT-qPCR

Quantification of differential gene expression was conducted and analyzed using the QuantStudio 7 and associated software (Applied Biosystems). qPCRs were generated using the TaqMan Fast Advanced Master Mix. All qPCRs were conducted in 384-well plates using 6 μl of an appropriate master mix (3.75 μl of TaqMan fast advanced master mix, 0.375 μl of TaqMan gene expression assay, and 1.875 μl of nuclease-free water) and 1.5 μl of either neat, 1:5, or 1:10 diluted cDNA for a total reaction volume of 7.5 μl. Each sample was run in triplicate for all genes of interest, and three wells of no template controls were included for every gene of interest to ensure no contamination of master mix or primers was present. Details of all primers and TaqMan gene expression assays used can be found in table S2. Analysis of differential gene expression was performed using the comparative CT method (*68*), using either *Gapdh* (hippocampus, striatum, and cortex), *Rps18* (lungs, heart, and aorta), or *Ywaz* (placenta) as a housekeeping control to normalize results. Data were expressed as a fold change relative to the relevant genotype PBS control group, which was normalized to one.

### Cytokine and chemokine protein expression

Circulating levels of cytokine and chemokines in blood were measured in duplicate using an 8-Plex Mouse Luminex Discovery Assay kit (R&D Systems, Minneapolis, MN, USA; catalog no. LXSAMSM-08) according to the manufacturer’s instructions. Analytes were quantified on a Bio-Plex 200 instrument (Bio-Rad, Gladesville, NSW, Australia) at 50 counts per region to measure median fluorescence intensity. Cytokine and chemokine concentrations were determined using a four-parameter fit standard curve.

### Blood pressure monitoring

Following infection, tail cuff plethysmography to measure the blood pressure of all mice was performed as previously described (*69*) using the BP-2000 Series II Blood Pressure Analysis System (Visitech Systems). Briefly, animals were placed in appropriately sized restrainers and secured onto the magnetic platform, preheated to 36°C. The noninvasive tail cuff was placed around the base of the tail, and the distal portion of the tail was secured onto the tail platform with adhesive tape below the photoplethysmography light and sensor. All animals underwent procedure acclimatization for blood pressure monitoring for 5 days starting at 1 dpi. At 6 dpi, 20 individual measurements of systolic, diastolic, and mean arterial pressure and pulse rate were recorded daily using the system’s integrated software. Any values from the 20 individual readouts that were beyond 2 SDs from the mean value were deemed as outliers and were excluded from analysis.

### Quantification of cardiac troponin III in plasma

The levels of cardiac troponin in the plasma of IAV-infected dams were quantified in plasma using the Mouse Troponin I Type 3 (cardiac) Sandwich ELISA Kit (Novus Biologicals), as per the manufacturer’s protocol. Plasma samples were run in duplicate, and optical density was read at 405 nm using the Clariostar Plus Plate Reader (BMG Labtech).

### Assessment of vascular function in dams

Aortic vascular function was determined by myography as previously described (*13*, *20*, *35*). The thoracic aorta was excised from dams at 6 dpi, and the PVAT was carefully removed under stereology microscopy. The cleaned vessel was trimmed to 2 mm in length and threaded carefully onto the two pins of a 610M Multi Myograph System (Danish Myo Technologies). The organ baths of the myograph were filled with 7 ml of a Krebs solution (119 mM NaCl, 4.7 mM KCl, 1.17 mM MgSO_4_, 25 mM NaHCO_3_, 1.18 mM KH_2_PO_4_, 5.5 mM glucose, and 2.5 mM CaCl_2_) prewarmed to 36°C and bubbled with a carbogen mixture (95% O_2_ and 5% CO_2_). Once mounted, vessels underwent an hour of acclimatization. Following acclimatization, all vessels were normalized to a resting tension of 5 mN. Maximum contraction was then established by a bolus administration of the thromboxane A2 agonist, U-46619 (10^−6^ M; Cayman Chemicals). After recording the maximal response, vessels were subjected to several washouts to restore baseline tension. U46619 was then titrated to achieve ~50% of the maximal contractile response, which served as the preconstriction level for subsequent relaxation assays. Endothelium-dependent vasorelaxation was assessed by cumulative addition of ACh (10^−9^ to 10^−5^ M), and vascular function was quantified as the percentage of relaxation relative to the preconstricted tone.

### Statistical analysis

All parametric data are expressed as the mean of the respective group ± the SEM as indicated by positive and negative error bars on all graphs. Nonparametric data are expressed as the median of the respective group and the 95% confidence interval. Statistically significant outliers were identified using the ROUT method (*Q* = 1%) and excluded from comparative analysis. Unless specified otherwise, comparisons made between experimental groups were conducted using ordinary two-way analysis of variance (ANOVA), three-way ANOVA, or mixed effects model analysis followed by Tukey’s post hoc test for multiple comparisons between experimental groups, and statistical significance was denoted where *P* ≤ 0.05 for parametric data. Maternal weight gain over gestation was analyzed using a two-way repeated measures ANOVA with Geisser-Greenhouse’s epsilon correction. Unless stated otherwise, symbols on graphs indicate post hoc multiple comparisons that were statistically significant (*P* ≤ 0.05). Multiple Mann-Whitney tests were used for comparisons between groups for nonparametric data, and significance was denoted where *P* ≤ 0.05. Pearson’s correlative analysis was conducted to determine the strength of correlation using the *r*^2^ value. An *r*^2^ value of 0.6 to 0.8 was considered to have a moderate correlation, and >0.8 was considered to have a strong correlation; the *P* value was reported (*70*, *71*). Litter size was controlled for as a random effect variable using the Nonlinear Mixed-Effects (nlme) package in RStudio (*72*). All statistical testing was conducted using GraphPad Prism (GraphPad Software; version 9.5.0) or R Statistical Software (version 4.5.1; R Core Team, 2025).
