# Supplementary material for: TLR7 alters the maternal immune landscape during influenza A infection to increase maternal and fetal morbidity
Source: Sci Adv. 2026 Apr 29;12(18):eady2382. doi: 10.1126/sciadv.ady2382 (PMC13127577; doi:10.1126/sciadv.ady2382)
Supplement: Supplementary file 1 — Figs. S1 to S10 Tables S1 and S2 [file sciadv.ady2382_sm.pdf]

Science Advances
AAAS

Supplementary Materials for

TLR7 alters the maternal immune landscape during influenza A infection to increase maternal and fetal morbidity

Gemma S. Trollope *et al.*

Corresponding author: Stavros Selemidis, stavros.selemidis@rmit.edu.au; Stella Liong, stella.liong@rmit.edu.au

Sci. Adv. **12**, eady2382 (2026)
DOI: 10.1126/sciadv.ady2382

This PDF file includes:

Figs. S1 to S10
Tables S1 and S2

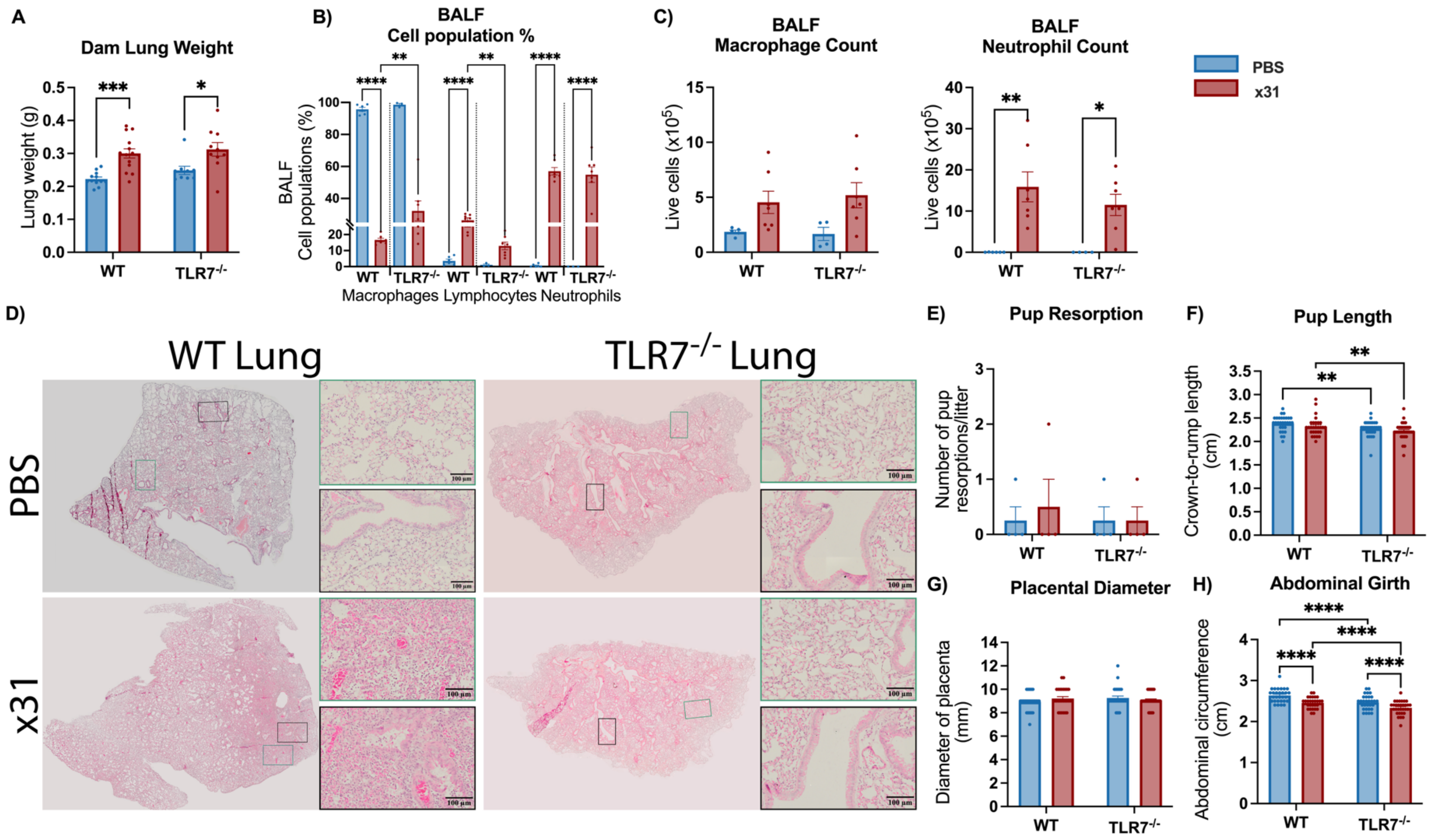

Fig. S1. TLR7 deficiency alters the pulmonary pathology of IAV infected pregnant mice. Lung and bronchioalveolar lavage fluid (BALF) was isolated from HK-x31 infected (x31; 10^4 PFU) or mock-infected (PBS) pregnant mice to assess pulmonary disease. **(A)** Gross weight of lungs isolated, **(B)** immune cell composition from differential staining in BALF and **(C)** total numbers of macrophages and neutrophils. **(D)** Representative images of H&E stained lungs used to score histopathology. Scale bar represents 100 µm. **(E)** Number of resorbed pups, **(F)** pup length, **(G)** placental diameter and, **(H)** pup abdominal girth. Statistical analysis was determined *via* Two-way ANOVA with Tukey's post-hoc test (*$p \leq 0.05$, **$p \leq 0.01$, ***$p \leq 0.001$, ****$p \leq 0.0001$). All data are presented as mean ± SEM. **(A)** n= 9-14 dams, **(B-C,E)** n= 4-7 dams per group, **(E-H)** n=28-31 offspring.

Cocktail 1

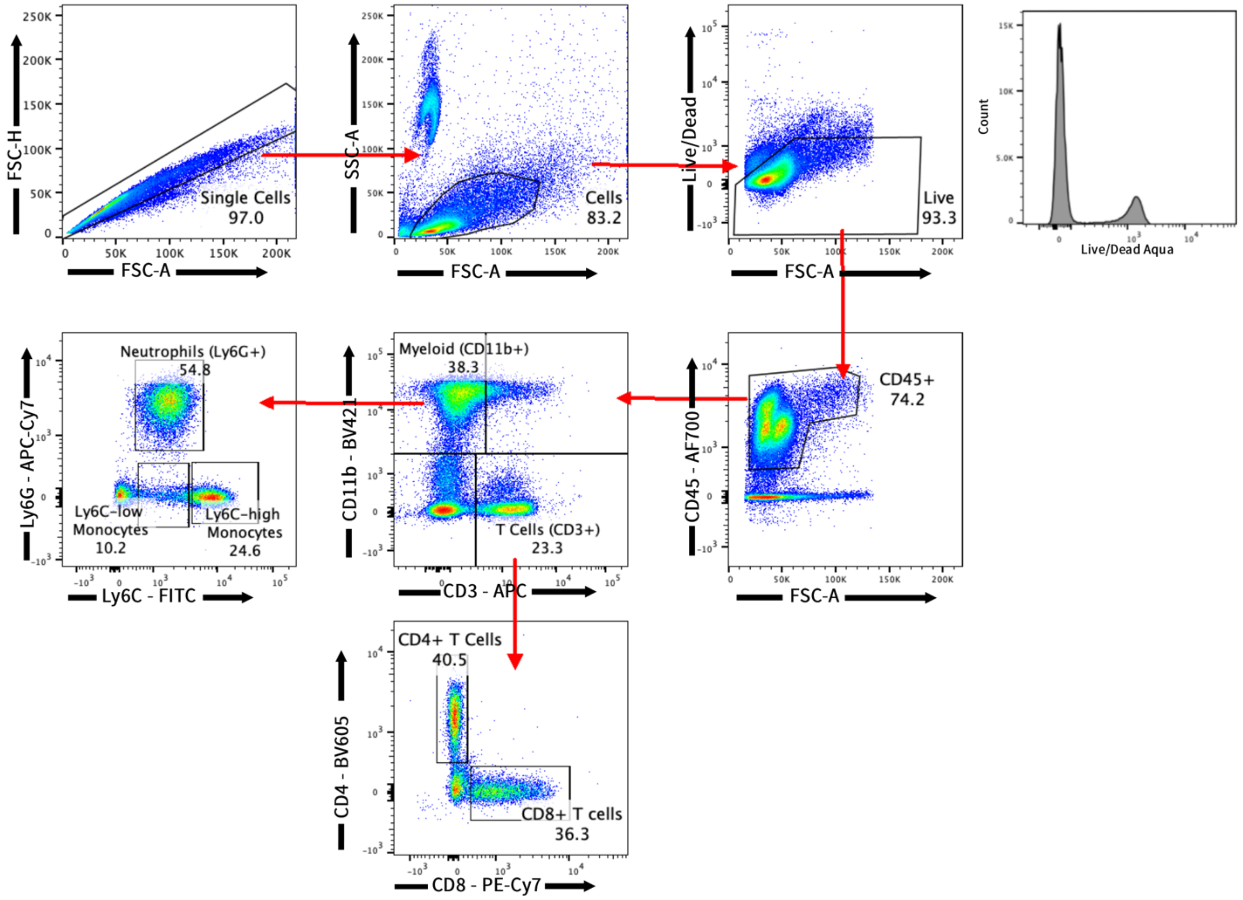

Cocktail 2

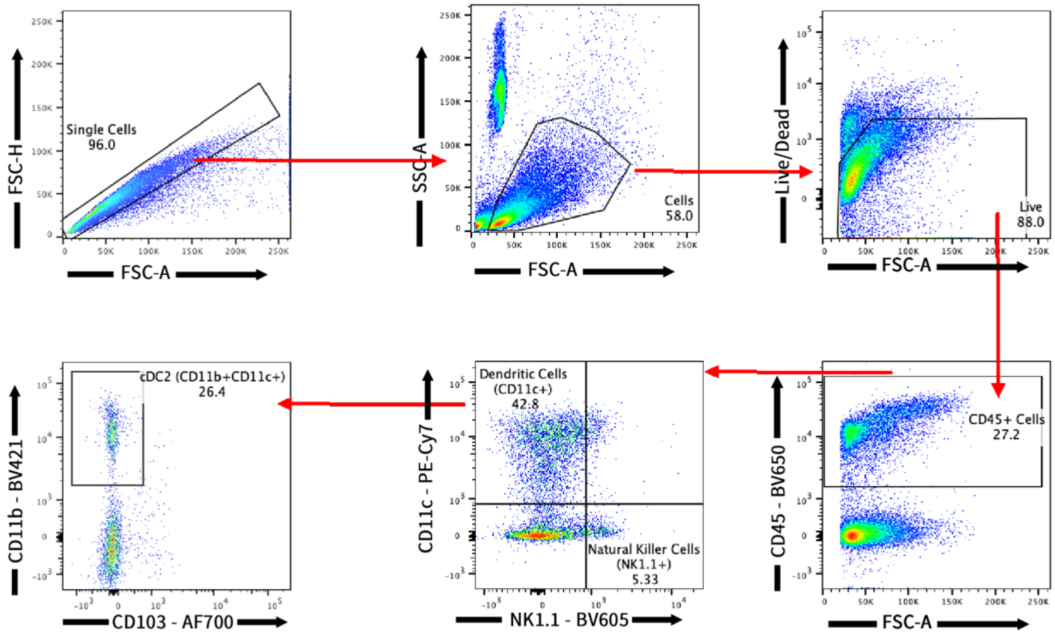

Fig. S2. Flow cytometry gating strategy for the phenotyping of immune cells within tissues isolated from IAV infected pregnant mice. The lungs, aorta, placenta, and decidua were stained with both cocktail 1 and cocktail 2, to determine the immunophenotype of the infiltrating cells in response to infection. Representative pseudocolor plots of flow cytometry data and a histogram indicating the staining pattern and intensity of non-viable cells. Cells are gated on singlets, then based on size and complexity to identify cells, negatively selected based on viability staining, then gated from $CD45^+$ cells to identify leukocytes. Individual immune cell populations were then confirmed based on expression of surface markers.

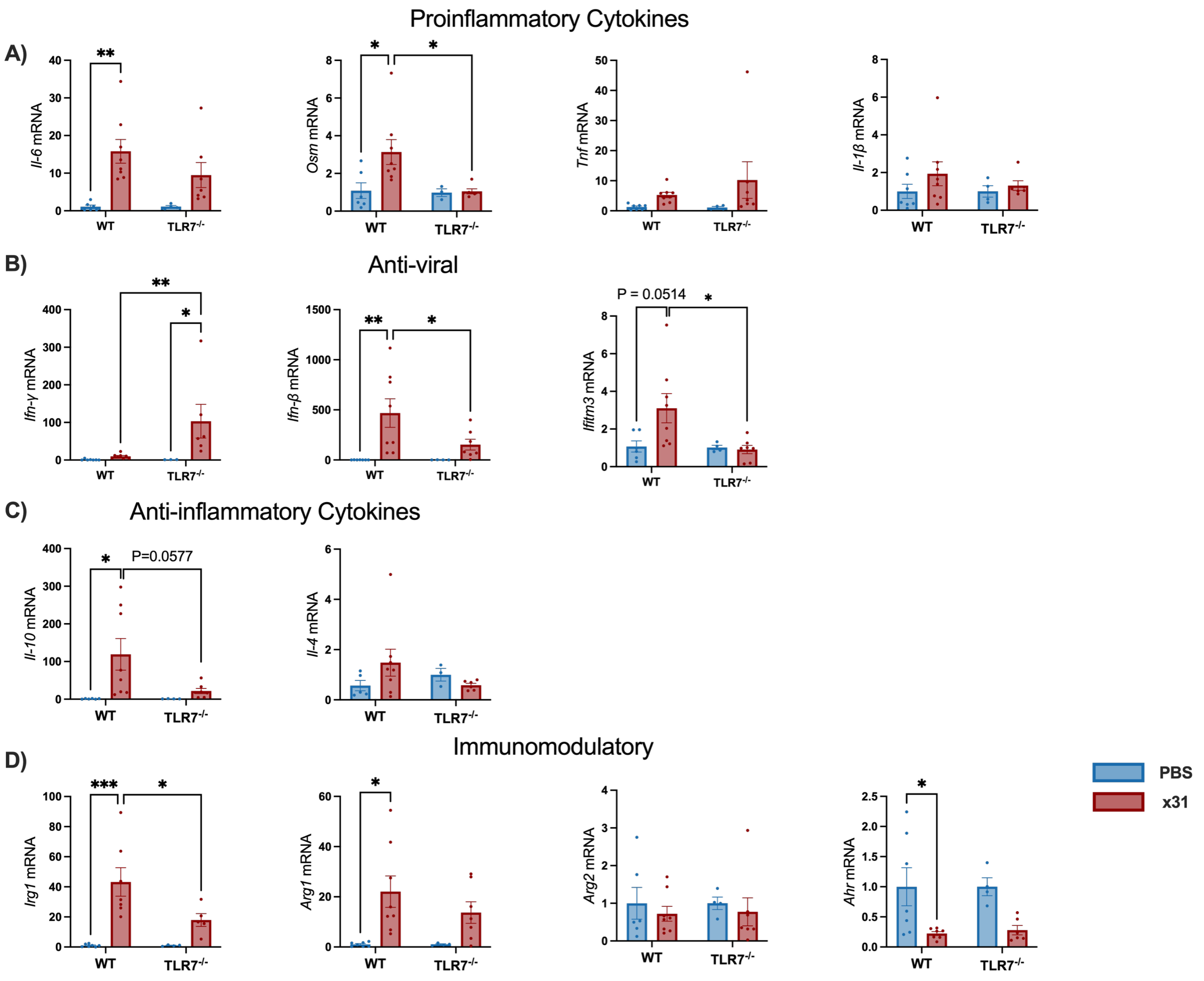
Proinflammatory Cytokines
A)
Il-6 mRNA
Osm mRNA
Tnf mRNA
Il-1β mRNA
B)
Anti-viral
Ifn-γ mRNA
Ifn-β mRNA
Ifitm3 mRNA
P = 0.0514
C)
Anti-inflammatory Cytokines
Il-10 mRNA
P=0.0577
Il-4 mRNA
D)
Immunomodulatory
Irg1 mRNA
Arg1 mRNA
Arg2 mRNA
Ahr mRNA
WT
TLR7-/-
PBS
x31

Fig. S3. Individual gene analysis from dam lungs. qPCR analysis of **(A)** proinflammatory cytokine, **(B)** anti-viral, **(C)** anti-inflammatory cytokine, and **(D)** immunomodulatory genes was normalized to the expression of Rps18 and expressed as the fold change (2-ΔΔCT method) of the geometric mean of the PBS controls. Statistical analysis was determined via Two-way ANOVA with Tukey's post-hoc test (*$p \leq 0.05$, **$p \leq 0.01$). All data are presented as mean ± SEM; n= 5-8 per group.

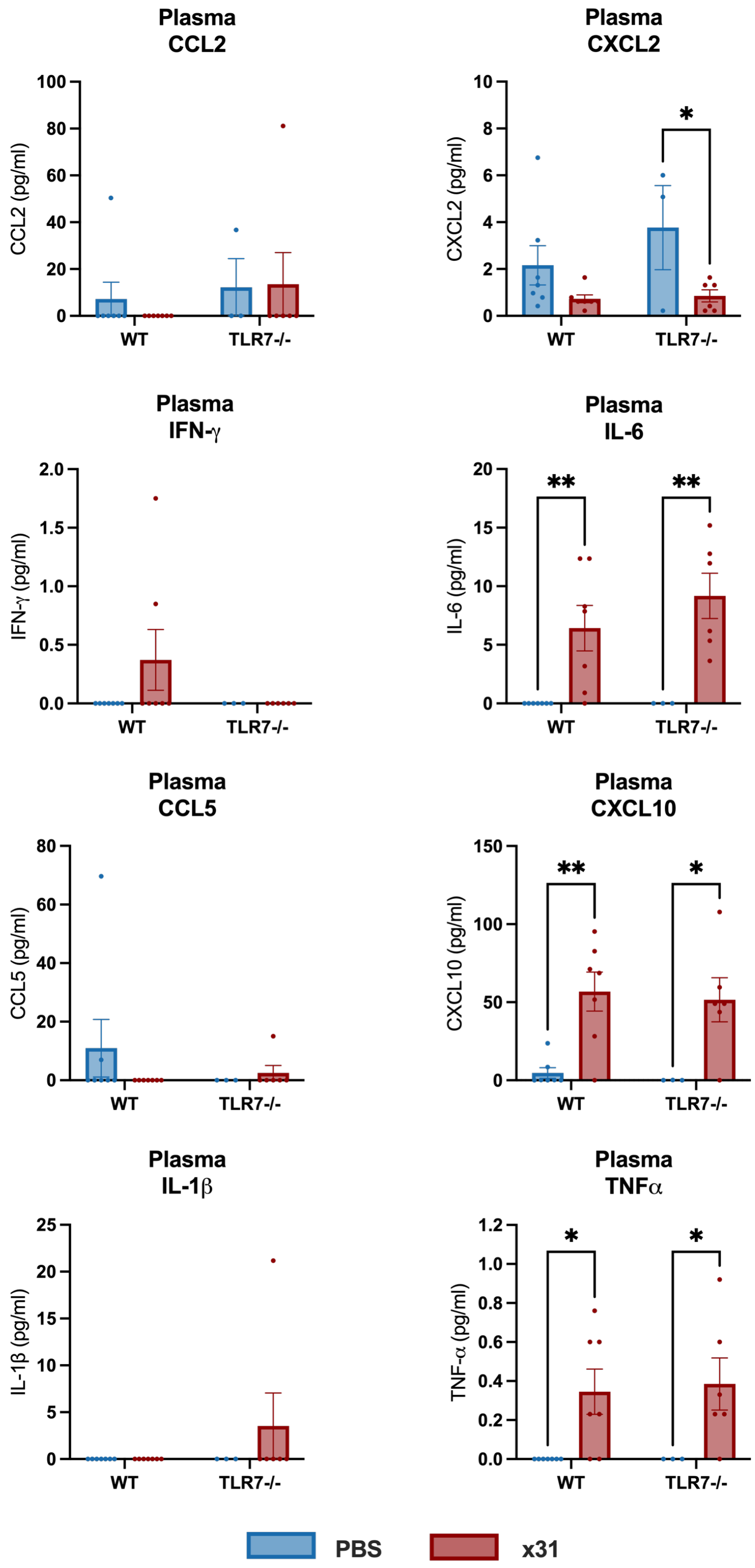
Plasma
CCL2
CCL2 (pg/ml)
WT
TLR7-/-
Plasma
CXCL2
CXCL2 (pg/ml)
Plasma
IFN-γ
IFN-γ (pg/ml)
Plasma
IL-6
IL-6 (pg/ml)
Plasma
CCL5
CCL5 (pg/ml)
Plasma
CXCL10
CXCL10 (pg/ml)
Plasma
IL-1β
IL-1β (pg/ml)
Plasma
TNFα
TNF-α (pg/ml)
PBS
x31

Fig. S4. Loss of TLR7 does not affect systemic inflammation in response to gestational IAV infection in pregnant dams. Systemic inflammation was assessed in pregnant WT and TLR7$^{-/-}$ mice infected with HK-x31 (x31; 10^4 PFU) or mock-infected (PBS). Cytokine and chemokine levels in plasma samples were assessed in x31- and mock-infected dams at 6 dpi. Statistical analysis was determined *via* Two-way ANOVA with Tukey's post-hoc test (*p≤0.05, **p≤0.01). All data are presented as mean ± SEM. n= 3-7 per group

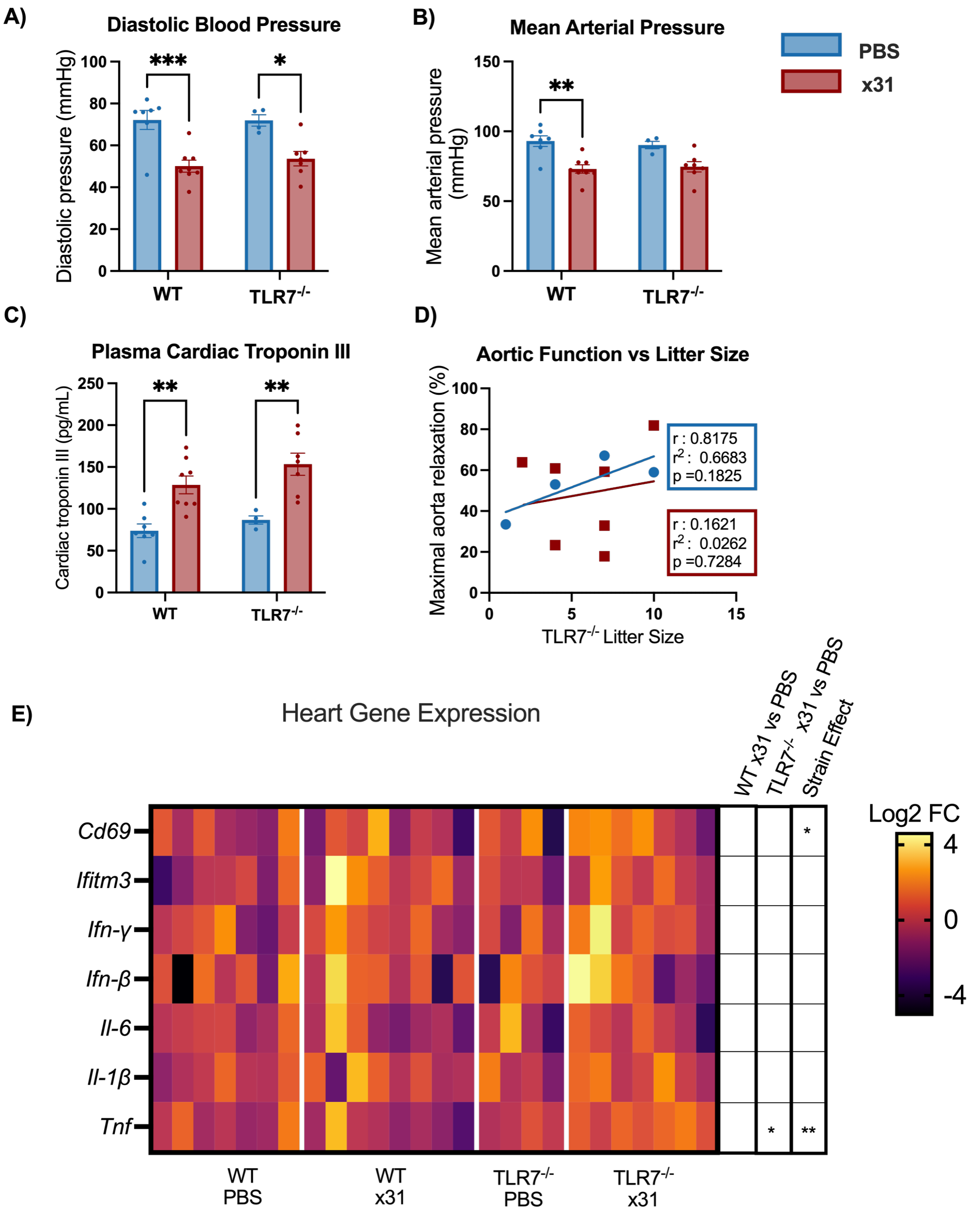

Fig. S5. Gestational IAV infection does not mediate inflammation in cardiac tissue of infected dams. Cardiovascular pathology was assessed in pregnant WT and TLR7$^{-/-}$ mice infected with HK-x31 (x31; 10^4 PFU) or mock-infected (PBS). **(A)** Diastolic blood pressure and **(B)** mean arterial pressure was measured over the course of 3 days (4-6 dpi) to determine the impact of IAV infection. **(C)** Cardiac troponin levels III in plasma were assessed to determine if

cardiac tissue damage was present and contributed to IAV induced bradycardia in dams. **(D)** The capacity for the aorta of pregnant mice were assessed vessel to relax in response to Acetylcholine (ACh) stimulation and was correlated with the size of the litter of TLR7$^{-/-}$ mice. **(E)** qPCR analysis of pro-inflammatory cytokine, anti-viral, T cell genes was normalized to the expression of *Rps18* and expressed as the binary logarithm (Log2) of the fold change of the geometric mean of the PBS controls. Statistical analysis was determined *via* Two-way ANOVA with Tukey's post-hoc test (*$p \leq 0.05$, **$p \leq 0.01$). All data are presented as mean ± SEM. n= 4-8 per group **(A-E)**.

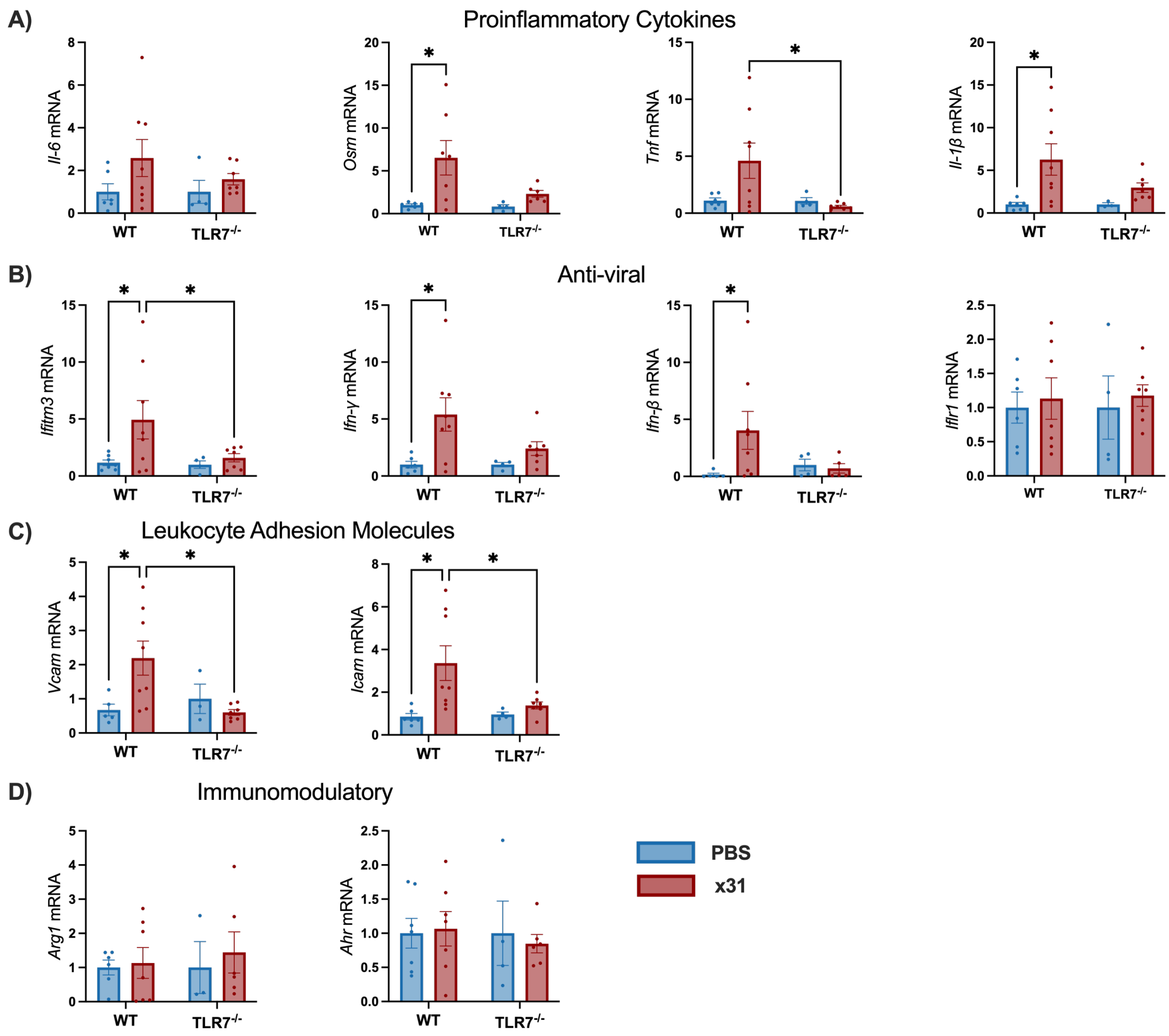

Fig. S6. Individual gene analysis from dam aorta. qPCR analysis of **(A)** proinflammatory cytokine, **(B)** anti-viral, **(C)** leukocyte adhesion, and **(D)** immunomodulatory genes was normalized to the expression of *Rps18* and expressed as the fold change ($2^{-\Delta\Delta CT}$ method) of the geometric mean of the PBS controls. Statistical analysis was determined *via* Two-way ANOVA with Tukey's post-hoc test (*p≤0.05). All data are presented as mean ± SEM; n= 5-8 per group.

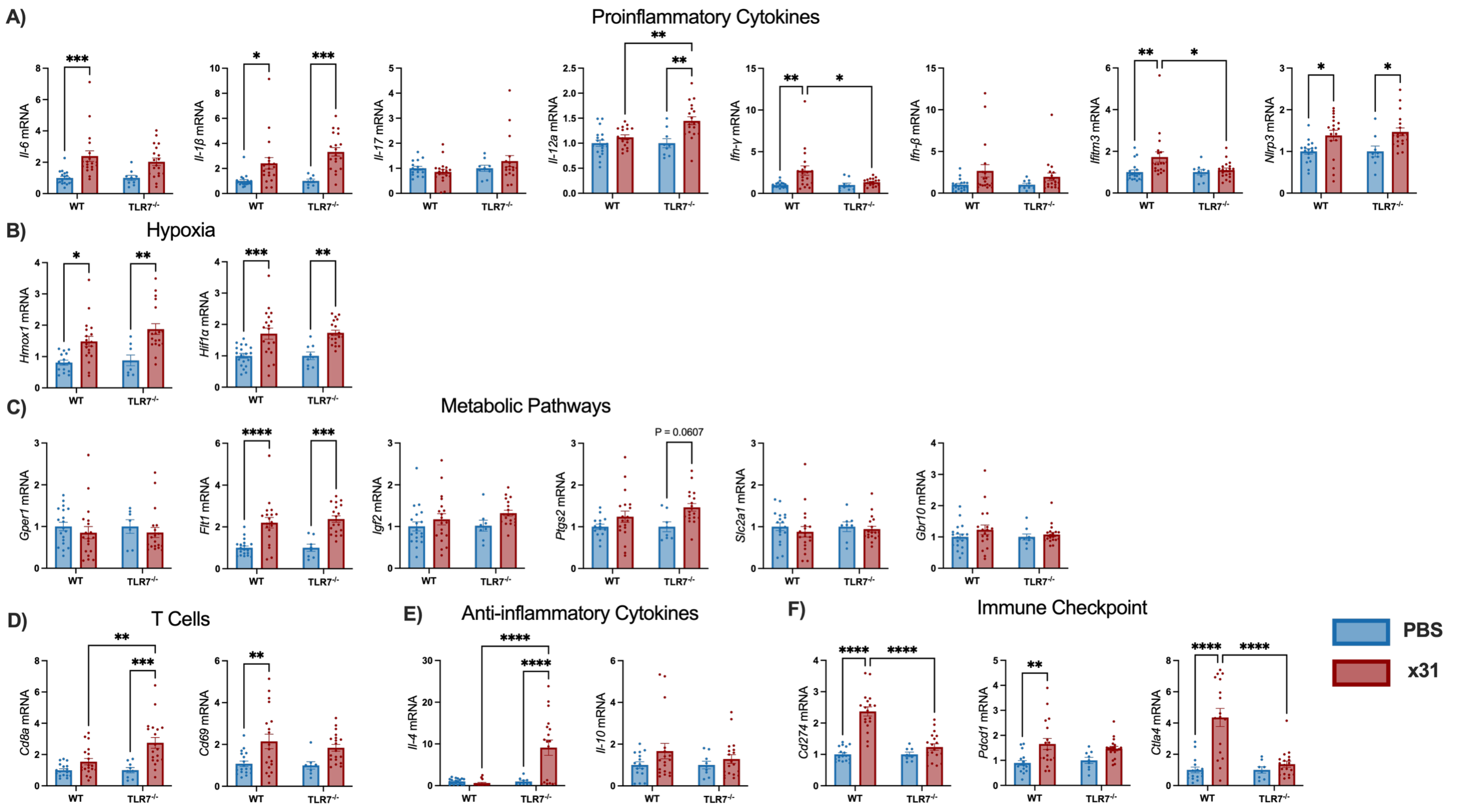

Fig. S7. Individual Gene Analysis from Placenta. qPCR analysis of **(A)** pro-inflammatory cytokine, **(B)** hypoxia, **(C)** metabolic pathway, **(D)** T cell, **(E)** anti-inflammatory cytokine, and **(F)** immune checkpoint genes was normalized to the expression of *Ywaz* and expressed as the fold change ($2^{-\Delta\Delta CT}$ method) of the geometric mean of the PBS controls. Statistical analysis was determined via Two-way ANOVA with Tukey's post-hoc test (*p≤0.05, **p≤0.01, ***p≤0.001, ****p≤0.0001). All data are presented as mean ± SEM; n= 9-19 per group.

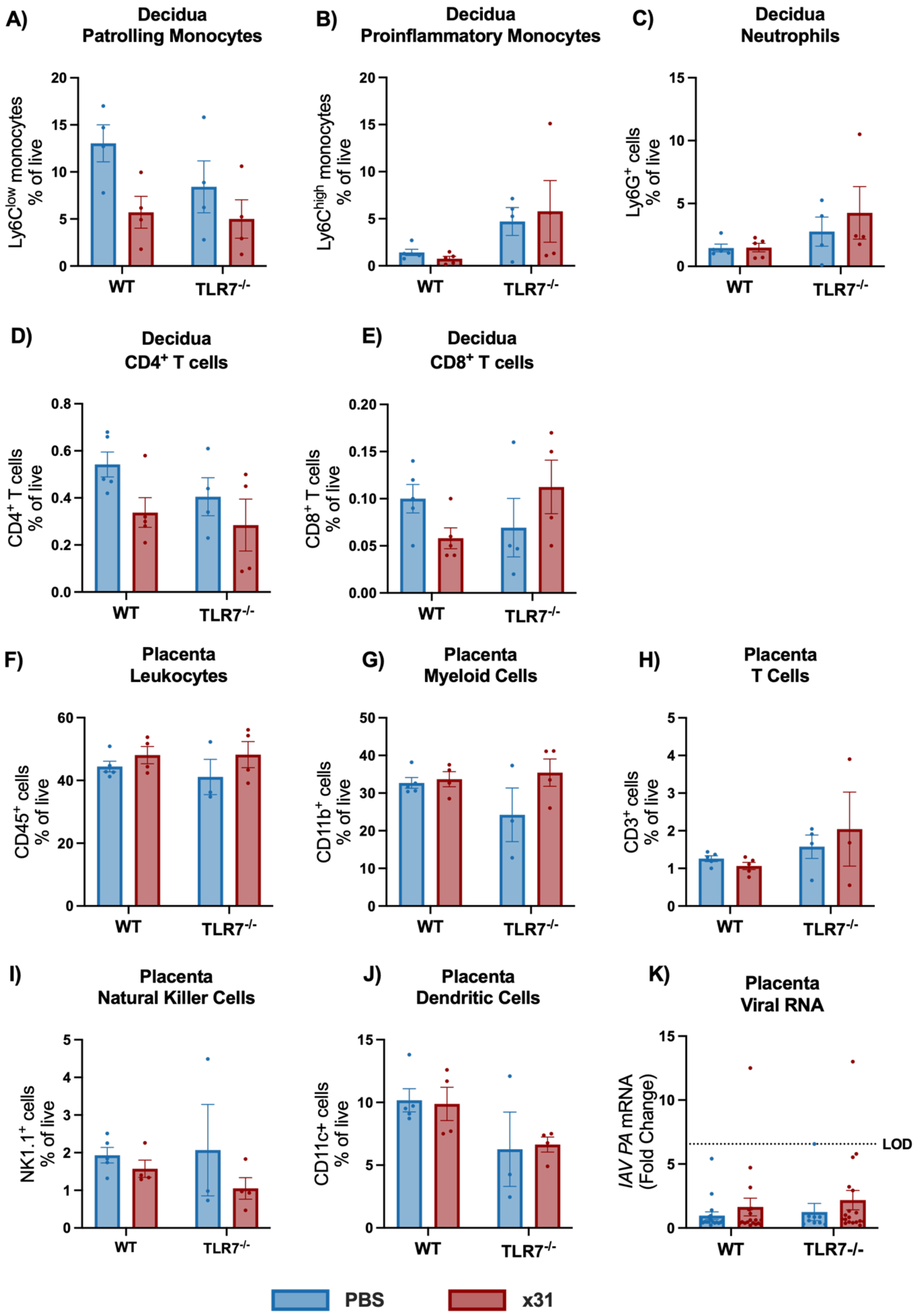

A) Decidua Patrolling Monocytes
Ly6Clow monocytes % of live
B) Decidua Proinflammatory Monocytes
Ly6Chigh monocytes % of live
C) Decidua Neutrophils
Ly6G+ cells % of live
D) Decidua CD4+ T cells
CD4+ T cells % of live
E) Decidua CD8+ T cells
CD8+ T cells % of live
F) Placenta Leukocytes
CD45+ cells % of live
G) Placenta Myeloid Cells
CD11b+ cells % of live
H) Placenta T Cells
CD3+ cells % of live
I) Placenta Natural Killer Cells
NK1.1+ cells % of live
J) Placenta Dendritic Cells
CD11c+ cells % of live
K) Placenta Viral RNA
IAV PA mRNA (Fold Change)
LOD
WT
TLR7-/-
PBS
x31

Fig. S8. IAV induced cell infiltration does not extend into the placenta from the maternofetal interface. Decidua and placentae of pregnant WT or TLR7$^{-/-}$ dams infected with Hk-X31 (x31; 104 PFUs) or mock infected with PBS were taken at 6 dpi and single cell suspensions were generated to investigate the changes in immune cell infiltration by flow cytometry **(A-J)**. Viral RNA was also quantified in placentae via qPCR, the limit of detection (LOD) is determined as the highest relative ratio value of uninfected (PBS) animals **(K)**. Data is presented as a proportion of live cells expressing the denoted marked marker gated on cell viability. Statistical analysis was determined via Two-way ANOVA with Tukey's post-hoc test (*p≤0.05, ***p≤0.001). All data are presented as mean ± SEM. n=4-5 per group **(A-J)** or n= 9-19 **(F)**.

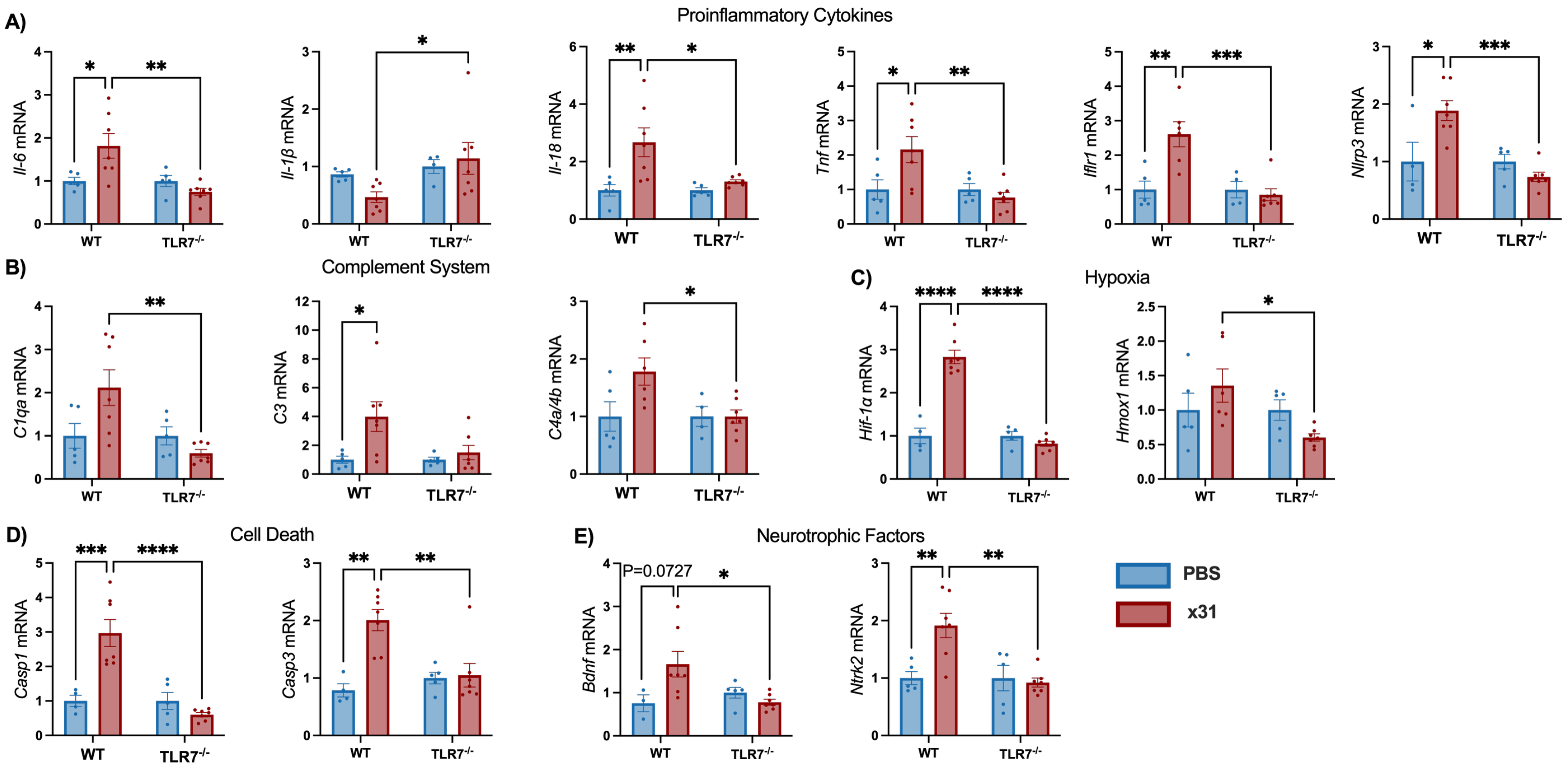

Fig. S9. Individual gene analysis from fetal hippocampus. qPCR analysis of **(A)** pro-inflammatory cytokine, **(B)** complement system, **(C)** hypoxia, **(D)** cell death, and **(E)** neurotrophic genes was normalized to the expression of *Gapdh* and expressed as the fold change ($2^{-\Delta\Delta CT}$ method) of the geometric mean of the PBS controls. Statistical analysis was determined via Two-way ANOVA with Tukey's post-hoc test (*p≤0.05, **p≤0.01, ***p≤0.001, ****p≤0.0001). All data are presented as mean ± SEM; n= 4-7 per group.

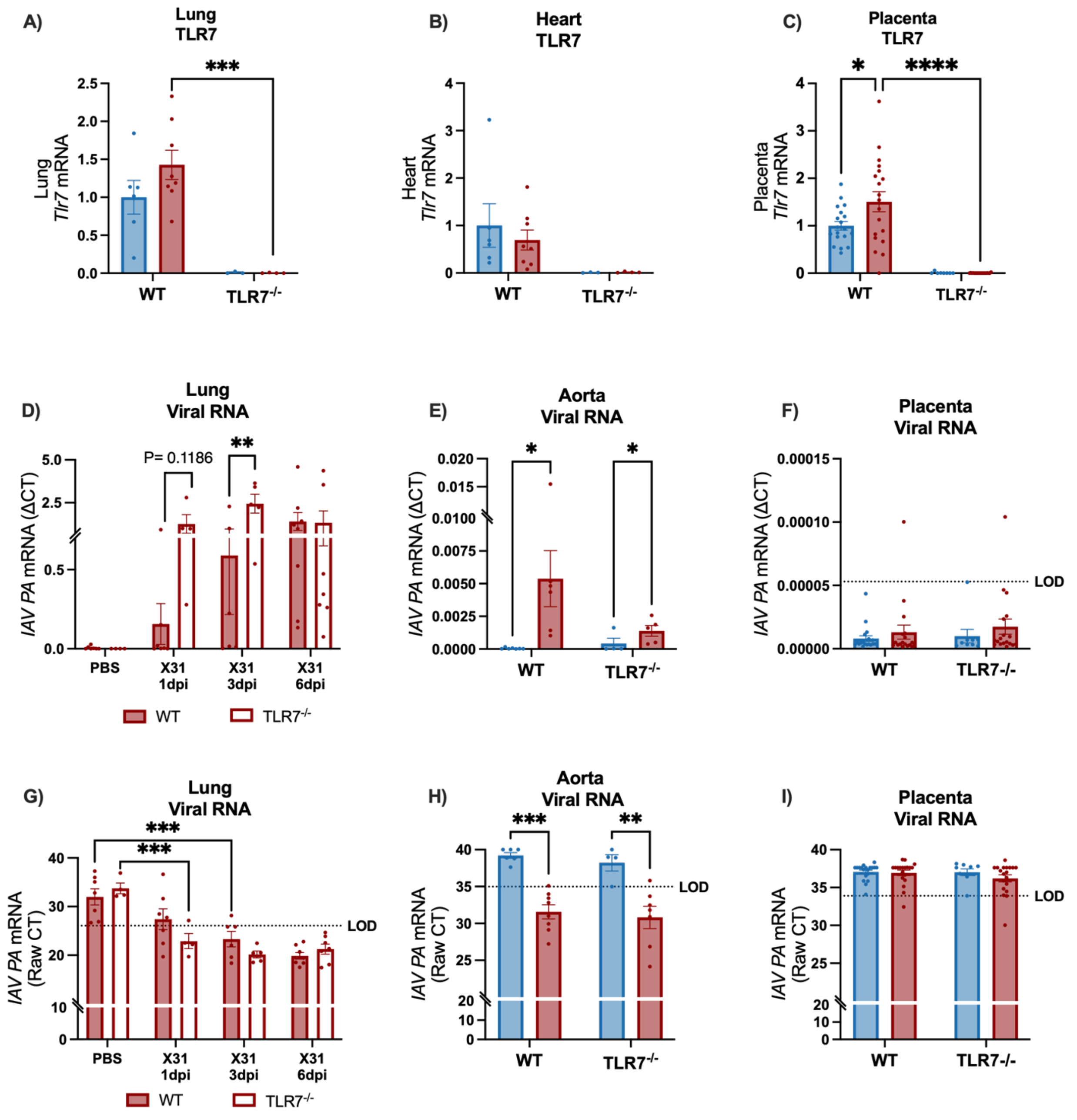

Fig. S10. Confirmation of TLR7$^{-/-}$ genotype and viral mRNA by qPCR in maternal and fetal tissues. qPCR analysis was conducted to confirm the complete knock out of TLR7 in maternal and fetal tissues. The expression of TLR7 mRNA was quantified in maternal **(A)** lung and **(B)** heart and **(C)** the placenta. Expression data was normalized to the housekeeping genes *Rps18* (lung and heart), or *Ywaz* (Placenta) and expressed as the fold change ($2^{-\Delta\Delta CT}$ method) of the geometric mean of the Wildtype PBS controls. Viral mRNA was quantified by qPCR in the lungs, aorta, and the placenta and expressed here as either anti-log ΔCT by normalising to the expression of *Rps18* (lung and aorta), or *Ywaz* (Placenta) **(D-F)**, or as the Raw CT value of the IAV *PA* gene **(G-I)**.Statistical analysis was determined via Two-way ANOVA with Tukey's

post-hoc test (*p≤0.05, **p≤0.01, ***p≤0.001, ****p≤0.0001). All data are presented as mean ± SEM; n= 4-7 per group **(A,B,D,E,G,H)** and n= 9-19 **(C,F,I)**.

Table S1. Summary of results for statistical difference in placental gene expression account for fetal sex. Gene expression was quantified via RT-qPCR. Statistical analysis was determined via 3-way ANOVA with mixed effect analysis. (*$p \leq 0.05$, **$p \leq 0.01$, ***$p \leq 0.001$, ****$p \leq 0.0001$). All data are presented as mean ± SEM; n= 7-11 per group

Gene	Strain	Infection	Sex	Strain x Infection	Strain x Sex	Infection x Sex	Strain x Infection x Sex
Il-6	ns	****	ns	ns	ns	ns	ns
Il-1β	*	****	ns	ns	ns	ns	ns
Ifn-γ	ns	**	ns	ns	*	ns	*
Ifn-β	ns	**	ns	ns	ns	ns	ns
Ifitm3	*	*	ns	ns	ns	ns	ns
Hmox1	ns	****	ns	ns	ns	ns	ns
Hif1α	ns	****	ns	ns	ns	ns	ns
Flt1	ns	****	ns	ns	ns	ns	ns
Ifg2	ns	*	ns	ns	ns	ns	ns
Nlrp3	ns	**	ns	ns	ns	ns	ns
Il-12a	**	**	ns	ns	ns	ns	ns
Il-4	*	**	ns	**	ns	ns	ns
Pdcd1	*	***	ns	ns	ns	ns	ns
Ctla4	***	****	ns	**	ns	ns	ns
Cd274	ns	****	ns	****	ns	ns	ns

Table S2. List of qPCR assays and primers. Details of Taqman gene expression assays and primers utilized in the quantification of mRNA in tissue samples, including Assay ID from Thermofisher Scientific and the corresponding GenBank accession number.

Target Gene	Assay ID/ Sequence	GenBank accession number	Target Gene	Assay ID/ Sequence	GenBank accession number
Ahr	Mm00478932_m1	AF405560.1	*Ifn-β*	Mm00439552_s1	K00020.1
Arg1	Mm00475988_m1	AK149471.1	*Ifn-γ*	Mm01168134_m1	AK089574.1
Arg2	Mm00477592_m1	AF032466.1	*Igf2*	Mm00439564_m1	BC053489.1
Bdnf	Mm04230607_s1	AK033127.1	*Il-10*	Mm00439614_m1	AK152344.1
C1qa	Mm00432142_m1	AK002655.1	*Il-12a*	Mm00434169_m1	AF128210.1
C3	Mm01232779_m1	BC029976.1	*Il-17*	Mm00439618_m1	AK040420.1
C4a/C4b	Mm00550309_m1	AK157954.1	*Il-18*	Mm00434226_m1	AY157834.1
Casp1	Mm00438023_m1	AK132826.1	*Il-1β*	Mm00434228_m1	AK168047.1
Casp3	Mm01195085_m1	AK080651.1	*Il-4*	Mm00445259_m1	AB174765.1
Cd274	Mm00452054_m1	AF233517.1	*Il-6*	Mm00446190_m1	AK089780.1
Cd69	Mm01183378_m1	AK017979.1	*Irg1*	Mm01224532_m1	AK036446.1
Cd8a	Mm01182107_g1	AK037467.1	*Nlrp3*	Mm00840904_m1	AF486632.1
Ctla4	Mm00486849_m1	AB097214.1	*Ntrk2*	Mm00435422_m1	AB377224.1
Flt1	Mm00438980_m1	D88689.1	*Osm*	Mm01193966_m1	AK155637.1
Gapdh	4352339E	AK002273.1	*Pdcd1*	Mm00435532_m1	AK039828.1
Gper1	Mm02620446_s1	AK018203.1	*Ptgs2*	Mm00478374_m1	AK049923.1
Grb10	Mm01180443_m1	AK030727.1	*Rps18*	Mm02601777_g1	AK050626.1
Hif-α	Mm00468869_m1	AF003695.1	*Slc2a1*	Mm00441480_m1	AK089353.1
Hmox1	Mm00516005_m1	AK150934.1	*Tlr7*	Mm00446590_m1	AF334942.1
Icam	Mm00516023_m1	AK149748.1	*Tnf*	Mm00443258_m1	AK153319.1
Ifitm3	Mm00847057_s1	AK003407.1	*Vcam*	Mm01320970_m1	AK016465.1
Iflr1	Mm00558035_m1	AK141338.1	*Ywhaz*	Mm03950126_s1	AK003664.1
IAV PA	F: CGGTCCAAATTCCTGCTGA<br>R: CATTGGGTTCCTTCCATCCA	JQ290184.1			
